# Supplementary material for: Pooled analysis of the association between mental health and violence against women: evidence from five settings in the Global South
Source: BMJ Open. 2023 Mar 14;13(3):e063730. doi: 10.1136/bmjopen-2022-063730 (PMC10030569; doi:10.1136/bmjopen-2022-063730)
Supplement: Author's manuscript [file bmjopen-2022-063730.draft_revisions.pdf]

BMJ Open

BMJ Open is committed to open peer review. As part of this commitment we make the peer review history of every article we publish publicly available.

When an article is published we post the peer reviewers' comments and the authors' responses online. We also post the versions of the paper that were used during peer review. These are the versions that the peer review comments apply to.

The versions of the paper that follow are the versions that were submitted during the peer review process. They are not the versions of record or the final published versions. They should not be cited or distributed as the published version of this manuscript.

BMJ Open is an open access journal and the full, final, typeset and author-corrected version of record of the manuscript is available on our site with no access controls, subscription charges or pay-per-view fees (http://bmjopen.bmj.com).

If you have any questions on BMJ Open's open peer review process please email info.bmjopen@bmj.com

BMJ Open

Pooled Analysis of the Association Between Mental Health and Violence Against Women: Evidence from five settings in the global South

Journal:	*BMJ Open*
Manuscript ID	bmjopen-2022-063730
Article Type:	Original research
Date Submitted by the Author:	11-Apr-2022

Complete List of Authors:	Ramsoomar, Leane; South African Medical Research Council, Gender and Health Research Unit<br>Gibbs, Andrew; South African Medical Association, Gender and Health Research Unit<br>Chirwa, Esnat; South African Medical Research Council,<br>Machisa, Mercilene T.; South African Med Res Council, Gender and Health Research Unit<br>Alangea, Deda; University of Ghana School of Public Health, Public Health<br>Addo-Lartey, Adolphina Addoley ; University of Ghana,<br>Dunkle, Kristin ; South African Medical Research Council,<br>Jewkes, Rachel; South African Medical Research Council, Gender and Health Division

Keywords:	MENTAL HEALTH, Depression & mood disorders < PSYCHIATRY, PUBLIC HEALTH, Substance misuse < PSYCHIATRY

SCHOLARONE™ Manuscripts

I, the Submitting Author has the right to grant and does grant on behalf of all authors of the Work (as defined in the below author licence), an exclusive licence and/or a non-exclusive licence for contributions from authors who are: i) UK Crown employees; ii) where BMJ has agreed a CC-BY licence shall apply, and/or iii) in accordance with the terms applicable for US Federal Government officers or employees acting as part of their official duties; on a worldwide, perpetual, irrevocable, royalty-free basis to BMJ Publishing Group Ltd ("BMJ") its licensees and where the relevant Journal is co-owned by BMJ to the co-owners of the Journal, to publish the Work in this journal and any other BMJ products and to exploit all rights, as set out in our licence.

The Submitting Author accepts and understands that any supply made under these terms is made by BMJ to the Submitting Author unless you are acting as an employee on behalf of your employer or a postgraduate student of an affiliated institution which is paying any applicable article publishing charge ("APC") for Open Access articles. Where the Submitting Author wishes to make the Work available on an Open Access basis (and intends to pay the relevant APC), the terms of reuse of such Open Access shall be governed by a Creative Commons licence – details of these licences and which Creative Commons licence will apply to this Work are set out in our licence referred to above.

Other than as permitted in any relevant BMJ Author's Self Archiving Policies, I confirm this Work has not been accepted for publication elsewhere, is not being considered for publication elsewhere and does not duplicate material already published. I confirm all authors consent to publication of this Work and authorise the granting of this licence.

For peer review only

Pooled Analysis of the Association Between Mental Health and Violence Against Women: Evidence from five settings in the global South

Leane Ramsoomar[1,2,3,] Andrew Gibbs[1,4], Esnat Chirwa[1,2], Mercilene Machisa[1,2], Deda Ogum Alangea[5], Adolphina Addo-Lartey[5], Kristin Dunkle[1], Rachel Jewkes[1,2]

1 Gender and Health Research Unit, South African Medical Research Council, Pretoria, South Africa

2 School of Public Health, University of the Witwatersrand, Johannesburg, South Africa

3 School of Public Health and Health Systems, University of the Pretoria, Gauteng, South Africa

4 Centre for Rural Health, School of Nursing and Public Health, University of KwaZulu-Natal, Durban, South Africa

5 School of Public Health, University of Ghana

Corresponding author:

Leane Ramsoomar [1,2,3] leane.ramsoomar@mrc.ac.za

[1] Gender and Health Research Unit, South African Medical Research Council, Pretoria, South Africa

[2] School of Public Health, University of the Witwatersrand, Johannesburg, South Africa

[3] School of Public Health and Health Systems, University of the Pretoria, Gauteng, South Africa

Abstract

Objectives: To describe associations between men's poor mental health (depressive and post-traumatic stress symptomatology) and their perpetration of Intimate Partner Violence (IPV) and non-partner sexual violence (NPSV), and women's mental health and their experiences of IPV and NPSV in five settings in the global South.

Design: A pooled analysis of data from baseline interviews with men and women participating in five Violence against Women and Girls (VAWG) prevention intervention evaluations.

Setting: Three Sub-Saharan African countries [South Africa (2), Ghana, and Rwanda], and one Middle Eastern country, the occupied Palestinian territories (oPt).

Participants: 7021 men and 4,525 women 18+ years old from a mix of self-selecting and randomly selected household surveys.

Main outcome measures: All studies measured depression symptomatology using the Centre for Epidemiological Studies-Depression (CESD), and the Harvard Trauma Scale for PTSD symptoms among

For peer review only

men and women. IPV and NPSV were measured using items from modified WHO Women's Health and Domestic Violence and UN Multi-country Study to assess perpetration among men, and experience among women.

Findings: Overall men's poor mental health was associated with increased odds of perpetrating physical IPV and NPSV. Specifically, men who had more depressive symptoms had increased odds of reporting IPV (aOR=2.13 [95%CI 1.58-2.87]) and NPSV perpetration (aOR= (1.62 95% CI 0.97 -2.71) compared to those with fewer. Men reporting PTSD had higher odds of reporting IPV (aOR= 1.87 [95% CI 1.44 -2.43]) and NPSV (aOR= 2.13 [95% CI 1.49 -3.05]) perpetration compared to those without PTSD. Women who had experienced IPV (aOR= 2.53 [95% CI 2.18 -2.94]) and NPSV (aOR= 2.65 [95% CI 2.02 -3.46]) had increased odds of experiencing depressive symptoms compared to those who had not.

Conclusions: Interventions aimed at preventing IPV and NPSV perpetration and experience must account for the mental health of men as a risk factor, and women's experience.

Article Summary

Strengths

- Synthesises comparable data across LMIC settings in the global South, thereby addressing the limited geographical scope of studies examining the association between mental health as a driver of men's violence perpetration against women, and as a consequence of violence experienced by women in the global South.
- Addresses both IPV and NPSV perpetration by men and experience by women using comparable measures.
- Highlights the need to integrate mental health focus into programmes aimed to prevent male to female intimate and non-intimate partner violence and interventions to address poor mental health outcomes among women experiencing IPV and NPSV in LMIC settings.

Limitations

- Only two of the five studies are population-based, limiting generalizability.
- All data are cross-sectional, limiting inference about causality and direction of effects.

For peer review only

Introduction

Violence against women and girls (VAWG), particularly, intimate partner violence (IPV) and non-partner sexual violence (NPSV) are significant global public health problems. Globally, about 35% of women report having experienced either physical and/or sexual intimate partner violence or non-partner sexual violence in their lifetime (1). A large body of research has emerged identifying drivers of men's perpetration of physical and or sexual violence against their female partners, and women's experience of this, in diverse contexts and populations. Drivers of violence perpetration and experience include poverty, gender inequalities, normalization of violence, substance use, exposure to abuse as a child, and poor communication in relationships and conflict skills, as well as living conflict-affected settings (2-5). Research has established associations between experience of IPV and a range of negative health outcomes, particularly mental health outcomes, HIV, low birthweight, termination of pregnancy, suicidal ideation and harmful alcohol use (1, 6).

Poor mental health is increasingly recognized as both an outcome and a driver of VAWG (7, 8). Among women, the associations between VAWG and negative health outcomes have been recognised for some time and poor mental health has largely been established as an outcome of IPV and NPSV experience (9). The WHO multi-country study on Women's Health and Domestic violence found significant associations between lifetime experiences of partner violence and self-reported poor health, including more emotional distress, suicidal thoughts and attempts among abused, compared with non-abused, women (2). A systematic review and meta-analysis of cohort studies found a strong association between recent IPV experience and depressive symptoms among women(10). Research has shown that poor mental health may also be a risk factor for women's IPV experience. For instance, Devries and colleagues found that women who experience depression may be more accepting of partners with traits that predispose them to violence (11), while other research found that women who experience depression may withdraw or display lethargy, which impacts their ability to seek help, or remove themselves from relationships (10). Furthermore, the systematic review of cohort studies (mentioned above), found evidence of a bi-directional relationship between depression and IPV experience, whereby women's depressive symptoms were associated with subsequent IPV experience(10).

Among men, research has found that poor mental health is associated with IPV perpetration, but the majority of this evidence has come largely from military or male offender samples from high income countries (12-14). There is, however, heterogeneity among samples and measures and definitions of IPV in previous work. Additionally this prior research has often focused on symptoms of PTSD (8, 13, 15, 16),

with evidence indicating strong associations between PTSD and IPV perpetration (13). Depression as a risk factor for male perpetration of violence against women has been less frequently studied and where examined, results have been mixed. For example, the cross-sectional population-based UN multi-country study in the Asia-Pacific region found that depression was associated with men's physical and sexual IPV perpetration in samples from four of six countries (17). However, evidence from a systematic review of population based studies found no association between depression and IPV perpetration (18). This may be due to limited numbers of population-based studies on men's violence perpetration, and the frequent omission of measures of men's mental health in studies of IPV perpetration. To our knowledge the association between men's poor mental health and their risk of NPSV perpetration has not been examined in LMICs.

Poor mental health often occurs against the backdrop of poverty, adverse childhood experiences, life traumatic events, low education and gender inequality and conflict and the wider context of generalized violence (3, 8, 19, 20). These factors operate either directly or indirectly to impact on childhood adverse events, poor mental health, substance use and relationship conflict, which in turn increase risk for IPV (3). Data from a population-based survey in South Africa found that experiencing adverse childhood events (physical, sexual and emotional abuse) increased the risk of men's poor mental health (PTSD, depression), harmful alcohol use, which in turn increased the likelihood of male-perpetrated IPV (8).

Although poor mental health has been recognized as both a driver and outcome of VAWG, the majority of studies are from high-income settings, focus largely on IPV only, and use differing measures of exposure and outcomes, which limits comparison across settings. There is much less research on these associations in low-middle income settings, where co-occurring public health challenges of VAWG, poor mental health and harmful alcohol use are prevalent. To strengthen the evidence base from low- and middle-income countries, and advance the current literature base on the role of poor mental health in both IPV and NPSV perpetration and victimisation, we undertook a pooled analysis of baseline data using comparable measurement methods from five IPV prevention studies conducted in three countries in Africa (South Africa – which had two studies - Ghana and Rwanda) and one conflict-afflicted country in the Middle East, the occupied Palestinian Territories (oPt). All studies report on recent (past 12 month) IPV and NPSV experience/perpetration, as opposed to lifetime exposure to IPV and NPSV. The paper aims to answer the following research questions (i) Is men's poor mental health associated with IPV and NPSV perpetration ? (ii) Is women's experience of IPV and NPSV associated with poor mental health outcomes?.

For peer review only

Methods

The studies included in this pooled analysis were conducted under the UK-Aid funded What Works to Prevent Violence Against Women and Girls? Global Programme (What Works). The primary goal of What Works was to advance the evidence base on the prevalence and drivers of VAWG in the Global South, and determine the effectiveness of interventions to prevent VAWG. The current study used the baseline data from 7021 men and 4,525 women from five VAWG prevention studies in four countries (South Africa, Ghana, Rwanda, and occupied Palestinian Territories) to assess the association between poor mental health (depression and PTSD symptomatology) and IPV and NPSV perpetration among men and the association between poor mental health and IPV and NPSV experience among women. These studies include the Stepping Stones and Creating Futures intervention (South Africa), Sonke Change trial (South Africa), Rural Response System (RRS-COMBAT) community intervention (Ghana), Indashyikirwa couples intervention (Rwanda), and a population-based nationally representative survey in the occupied Palestinian Territory (oPt). Further information on the studies is available in the study sources referenced in Table 1.

Patient and public involvement

Patients and the public were not involved in the study design, implementation, or analysis presented here. However, the individual projects had different levels of participant and public involvement as part of the wider What Works research uptake strategy.

For peer review only

Table 1: **Datasets used for men and women included in the pooled analysis**

#	Study	Country	Study Design	# clusters	N (Men)	N (Women)	Sampling or recruitment strategy	Age
1	Evaluation of Stepping Stones and Creating Futures	South Africa	CRCT	34	674	677	Study volunteers	18-35
2	Evaluation of Sonke Change trial	South Africa	CRCT	18	2406	-	Household-based random sample survey	18-45
3	Evaluation of the RRS-COMBAT community intervention	Ghana	CRCT	40	1973	1877	Household-based random sample survey	18+ (men) 18-45 (women)
4	Indashyikirwa -- couples intervention	Rwanda	CRCT	28	1651	1660	Volunteer recruitment from savings and loan association groups	18-50
5	Evaluation of Innovative Media to End VAWG through Community Education and Outreach in the oPt	Occupied Palestinian Territories	Population-based survey	55	308	371	Population-based nationally representative survey	18+ (men) 18+ (women)

For peer review only

Measures

All study measures are presented in Table 2. We assessed socio-demographics for men and women, including, age, current marital status, relationship residence status, and education, employment in the past 3 months and past year, respectively. All studies except Indashyikirwa (couples) asked whether participants had worked in the past 3-months or the past year respectively; the two South African studies asked whether female participants had worked in the past 3-months or not. Income assessments in Indashyikirwa couples were not included in the present analyses, as they were tailored to the local context in which most participants engaged in subsistence agriculture.

Depression was measured using the previously validated Center for the Epidemiological Studies of Depression Short Form (CESD-10) (21, 22). PTSD was measured in three of the five studies (SSCF, Sonke Change trial, Ghana (men only), using the previously validated Harvard Trauma questionnaire (23), in settings where it was anticipated that the intervention would impact it. Men's current alcohol use was assessed by asking one question about alcohol use in the 12 months preceding the baseline data collection, while women were asked if they had seen their partner drunk, and how frequently they saw them drunk in the past 12 months. Violence perpetration (IPV and NPSV) and experience (IPV) were measured using items from the WHO Women's Health and Domestic Violence survey (24), modified appropriately to assess men's perpetration (17). The NPSV scale was first developed in South Africa (25) and subsequently refined and used extensively in the Asia-Pacific region (26). There was no question on NPSV perpetration in the Indashyikirwa couples or the oPt.

For peer review only

For peer review only - http://bmjopen.bmj.com/site/about/guidelines.xhtml

Table 2: **Key Measures**

**CONSTRUCT**	**Indicator**	**Definition**
**VIOLENCE AGAINST WOMEN**	Physical IPV perpetration	Five items were used to measure men's physical IPV perpetration including: In the last 12 months how many times (1) did you slap your current or previous girlfriend or wife or throw something at her which could hurt her? (2) have you pushed or shoved a current or previous girlfriend or wife? (3) have you hit a current or previous girlfriend or wife with a fist or with something else which could hurt her? (4) did you kick, drag, beat, choke or burn a previous or current girlfriend, partner or wife? (5) did you threaten to use or actually use a gun, knife or other weapon against a previous or current girlfriend, partner or wife? Responses: "Never", "Once" "A few times" or "Many times". Men who responded "Once" or more to one or more items were coded as perpetrating physical IPV. The items were developed during the WHO Women's Health and Domestic Violence survey, and UN Multi-country study on Men and Violence in Asia and the Pacific, modified to assess men's perpetration of physical and NPSV in the past year(17, 24).
	Physical IPV experience	Five items used to capture women's violence experience including: In the past 12 months, (1) how many times has a current or previous husband (or boyfriend) slapped you or thrown something at you which could hurt you? (2) pushed or shoved you? (3) hit you with a fist or with something else which could hurt you? (4) kicked, dragged, beaten, choked or burnt you? (5) threatened to use, or actually used, a gun, knife or other weapon against you?(24) Responses: "Never", "Once" "A few times" or "Many times". Women who responded "Once" or more to one or more items were coded as experiencing physical IPV.
	NPSV perpetration	Five items about NPSV perpetration, including: In the last 12 months how many times have you: (1) forced or persuaded a woman or girl who was not your girlfriend or wife at the time to have sex with you? (2) ever tried to force or persuade any woman or girl who was not your girlfriend or partner to have sex with you,

For peer review only - http://bmjopen.bmj.com/site/about/guidelines.xhtml

		but did not succeed? (3) ever had sex with a woman or girl who was not your girlfriend or wife when she was too drunk or drugged to stop you? (4) have you and other men ever had sex with a woman or girl at the same time who was not your girlfriend or wife when she did not agree to sex or you forced her? (5) have you and other men ever had sex with a woman or girl at the same time who was not your girlfriend or wife when she was too drunk or drugged to stop you?<br>Responses: Never", "Once" "A few times" or "Many times". Men who responded "Once" or more to one or more items were coded as perpetrating NPSV. The scale was first developed in South Africa and subsequently refined and used extensively in Asia-Pacific region(26).
**ALCOHOL USE**	Current Alcohol Use (past 12 months)	Current alcohol use by men was measured by asking one question about alcohol use in the 12 months preceding the baseline data collection: (1) Have you drunk alcohol in the past 12 months?. Responses were either "Yes" or "No".
	Seen partner drunk	1 item assessing if women has seen their partner drunk in the past 12 months. Responses: "Yes" or "No"
**MENTAL HEALTH**	Depression (men and women)	Three studies (SSCF, Sonke Change Trial and RRS-COMBAT) used the 20-item Centre for Epidemiological Studies Depression (CESD) scale used to measure depression. Items were framed around statements about feelings in the past week, such as: 'During the past week I felt fearful' with responses, 'none or rarely', 'some or a little', 'moderate amount of time', and 'most or all the time'. Items were summed, and ranged between 0-41, with higher scores indicating more depressive symptoms (Cronbach alpha ranged from 0.78-0.91 across studies). Three studies (Indashyikirwa and oPt) used the 10-item Centre for Epidemiological Studies Depression (CESD) short form scale to measure depression. Items were summed, and ranged between 0-21, with higher scores indicating more depressive symptoms.
	Post-Traumatic Stress Disorder (PTSD) symptoms (men and women).	Harvard Trauma Scale for PTSD, which reflected reactions to traumatic experiences in their lifetime experienced in the past week, such as: " In the past week have you had recurrent thoughts or memories of the most hurtful or terrifying events" or " . In the past week have you had recurrent nightmares". Responses were rated 0= not at all, to 3= extremely. Items were summed with higher scores indicating more PTSD symptoms. Cronbach alphas ranged from 0.91-0.94 across studies where PTSD was measured.

For peer review only - http://bmjopen.bmj.com/site/about/guidelines.xhtml

Data analysis

Descriptive statistics (frequencies and percentages) were used to summarize participants' socio-demographic characteristics within each study and in the pooled analysis. Within-study and pooled estimates considered any clustering with each study's sampling procedures. All pooled estimates were weighted according to the study sample size. We used forest plots, I-square and Cochran's Q statistics to assess the consistency of outcomes across the studies. The I-square values showed high heterogeneity in physical IPV as an outcome (80%, p-value<0.001 for men) and low heterogeneity for depression among women (14.8%, p-value=0.329). We used mixed-effects models to estimate overall effects and account for any heterogeneity across the studies due to methodological diversity. We fitted a one-stage Individual Patient Data (IPD) meta-analysis using mixed-effects logistic regression models to account for within-and between-study variances (heterogeneity) across studies for both men and women (17). We derived study-specific estimates and forest plots from a post-estimation model of the mixed-effects logistic regression model. Both the main and post-estimation models included participants' age as fixed effects. All data were analyzed using Stata Statistical Software: Release 17. College Station, TX: StataCorp LLC. StataCorp. 2019 and all models were adjusted for participants' age and alcohol use or partner alcohol use (for women), because of the association between age and IPV experience/perpetration, and co-morbidity between alcohol, VAWG and poor mental health found in previous research.

Results

Men

The mean age of men across all studies was 33.1 years old (SD=11.4). Forty-five percent of men were married while 45% were in a non-marital relationship. Overall, only 10% of men across all studies were not in a relationship; 61% of men indicated they were living with their partners, except in Rwanda (100%) couples and oPt (95%) where the majority were married. Almost a third of men (29%) reported not living together with their female partners. Most of the men had secondary school education or above (66%) and half (53%) the men in three studies were employed in the past 3 months (we did not ask about employment status in Rwanda and the oPt). (Table 3)

Women

The mean age of women across the four studies was 31.3 years old (SD=8.4). Over half (56%) of women were married, 38% were in a relationship, 75% were living with their partner or spouse and 19% were not living with their partner, and 6% were not in a relationship. Nearly one-half (48%) of the women had

For peer review only - http://bmjopen.bmj.com/site/about/guidelines.xhtml

completed secondary school education or above, and 53% reported working in the past 3 months, though this was not asked in Rwanda or oPt. Just over a third of women (32%) reported that their partner consumed alcohol in the past month. (See Table 3)

For peer review only

For peer review only - http://bmjopen.bmj.com/site/about/guidelines.xhtml

Table 3: **Socio-demographics, prevalence of alcohol consumption among men, reports of partner alcohol consumption among women in the included studies**

	Stepping Stones and Creating Futures Trial, South Africa	Sonke Change Trial, South Africa	RRS COMBAT, Ghana	Indashyikirwa - couples, Rwanda	oPt (Palestine)	All studies
	n(%) /mean(sd)	n(%)/mean(sd)	n(%) /mean(sd)	n(%) /mean(sd)	n(%) /mean(sd)	n(%) /mean(sd)
**MEN**	**(n=674)**	**(n=2406)**	(n=1973)	(n=1651)	(n=317)	**(n=7021)**
**Age**	23.8(3.6)	27.6(5.7)	39.3(14.9)	35.6(7.1)	43.9(13.8)	33.1(11.4)
**Current marital status**						
Married	22(3.3)	448(18.6)	1271(64.4)	1095(66.3)	301(95.0)	3137(44.7)
In a relationship	508(75.4)	1539(64.0)	533(27.0)	556(33.7)	0(0)	3136(44.7)
No relationship	144(21.4)	391(16.2)	169(8.6)		16(5.0)	720(10.3)
missing	0(0)	28(1.2)	0(0)			28(0.4)
**Relationship residence status**						
Living together	73(10.8)	932(38.7)	1314(66.6)	1651(100)	301(95.0)	4271(60.8)
Not living together	457(67.8)	1055(43.9)	490(24.8)		0(0)	2002(28.5)
No relationship	144(21.4)	391(16.2)	169(8.6)		16(5.0)	720(10.3)
missing	0(0)	28(1.2)	0(0)			28(0.4)
**Education**						
none	0(0)	0(0)	372(18.9)	265(16.1)	14(4.4)	651(9.3)
Primary school	77(11.4)	140(5.8)	334(16.9)	1088(65.9)	75(23.7)	1714(24.4)
Sec school or above	597(88.6)	2249(93.5)	1267(64.2)	298(18)	227(71.6)	4638(66.1)
missing	0(0)	17(0.7)	0(0)		1(0.3	18(0.3)
**Employed in past 3 months**	240(35.7)	1192(50)	1231(71.6)	n/m*	n/m	2663(52.7)
**Employed in past year**	156(23.2)	806(33.7)	1251(63.4)	n/m	n/m	2213(43.8)
**Drinks alcohol**	294(43.6)	948(39.4)	291(15.0)	246(15.0)	n/m	1779(27.0)

For peer review only - http://bmjopen.bmj.com/site/about/guidelines.xhtml

WOMEN	(n=677)	-	(n=1877)	(n=1660)	(n=371)	(n=4585)
**Age in yrs – mean(sd)**	23.9(3.6)		31.4(8.5)	32.7(6.6)	37.7(11.7)	31.3(8.4)
**Current marital status**						
Married	29(4.28)	-	1068(56.9)	1096(66)	365(98.4)	2558(55.8)
In a relationship	524(77.4)	-	664(35.4)	564(34)	0(0)	1752(38.2)
No relationship	124(18.3)	-	145(7.7)	0(0)	6(1.6)	275(6)
**Relationship residence status**						
Living together	113(16.7)	-	1303(69.4)	1660(100)	365(98.4)	3441(75.1)
Not living together	440(65.0)	-	429(22.9)	0(0)	0(0)	869(19)
No relationship	124(18.3)	-	145(7.7)	0(0)	6(1.6)	275(6)
**Education**						
None	0(0)	-	401(21.4)	288(17.4)	3(0.8)	692(15.1)
Primary school	56(8.3)	-	426(22.7)	1115(67.2)	120(32.4)	1717(37.5)
Sec school or above	621(91.7)	-	1050(55.9)	257(15.5)	248(66.8)	2176(47.5)
**Employed in past 3 months**	173(25.6)	-	1174(62.7)	n/m	n/m	1347(52.8)
**Employed in past year**	97(14.3)	-	1034(55.1)	n/m	n/m	1131(44.3)
**Seen Partner frequently drunk in past year**	357(52.7)		268(14.3)	742(44.7)	n/m	1367(32.4)
Never	320 (47.3)	-	1609 (85.7)	918 (55.3)	n/m	2847
Occasionally	220 (32.5)	-	115 (6.1)	471 (28.4)	n/m	806
Frequently	137 (20.2)	-	153 (8.2	271 (16.3)	n/m	561

* not measured

For peer review only - http://bmjopen.bmj.com/site/about/guidelines.xhtml

IPV and NPSV perpetration, poor mental health and alcohol consumption among men

Among the 7,021 men across the studies, almost a third (29%) reported perpetrating past year intimate partner violence, with study-specific prevalence ranging from 11% (Ghana) to 50% (SSCF). Across the three studies where men were asked about perpetration of NPSV, 26% of men reported perpetrating past year NPSV, ranging from 10% in Ghana to 39% in SSCF. We did not ask about the perpetration of NPSV in Rwanda or oPt as this was not the objective of these interventions. Overall, 29% of men reported depressive symptoms (range: 18% to 46%), and in the three studies where we asked about PTSD symptomatology (SSCF, Sonke Change trial and Ghana), 6% reported PTSD (range: 5-14.2%). In four studies where alcohol was asked about, 68% of men reported alcohol consumption in the past year (SSCF); 64% in the Sonke Change Trial; 44% in RRS-COMBAT, and 37% in Indashyikirwa. Alcohol consumption was not included in the questionnaire in the oPt. (See Table 4)

Women

IPV and NPSV experience, poor mental health, and reports of partner alcohol consumption

Among the 4,585 women across the studies, just over a third (31%) reported experiencing past year intimate partner violence, with study-specific prevalence ranging from 16% (Ghana) to 60% (SSCF). Across the two studies where NPSV was reported (SSCF and RRS-COMBAT), 11% of women reported experiencing past year NPSV, ranging from 3% in Ghana to 34% in SSCF. Overall, 36% of women reported depressive symptoms (range: 26% to 45%). Only women in SSCF were asked about PTSD symptomatology, and 21% reported such symptoms. (See Table 4)

For peer review only - http://bmjopen.bmj.com/site/about/guidelines.xhtml

Table 4: **Prevalence of Depression, PTSD symptomatology, Physical IPV and NPSV perpetration among men and experience among women in the studies included**

		Depression	PTSD	Physical IPV	NPSV
**MEN**					
	**N**	**n(%)**	**n(%)**	**n(%)**	**n(%)**
SSCF-SA	674	313(46.4)	96(14.2)	337(50.0)	261(38.7)
Sonke-SA	2406	700(29.1)	126(5.0)	952(39.6)	834(34.7)
RRS COMBAT-Ghana	1973	619(31.4)	71(3.60)	235(11.9)	196(9.9)
Indashyikirwa-Rwanda	1651	290(17.6)	n/m	402(24.4)	n/m*
oPt-Palestine	308	112(35.3)	n/m	73(23.0)	n/m
**All**	**7021**	**2034(29.0)**	**293(5.8)**	**1999(28.5)**	**1291(25.5)**
**WOMEN**					
	**N**	**Depression**	**PTSD**	**Physical IPV**	**NPSV**
SSCF-SA	677	306(45.2)	142(21.0)	403(59.5)	228(33.7)
RRS COMBAT-Ghana	1877	695(37.0)	*n/m	290(15.5)	54(2.88)
Indashyikirwa-Rwanda	1660	429(25.8)	n/m	629(37.9)	n/m
oPt-Palestine	371	201(54.2)	n/m	94(25.3)	n/m
All	**4585**	**1631(35.6)**	**142(21.0)**	**1416(30.9)**	**282(11.0)**

* not measured

For peer review only - http://bmjopen.bmj.com/site/about/guidelines.xhtml

Men

Association between poor mental health and men's violence perpetration

The association between men's depressive symptomatology and perpetration of past year IPV was significant across four studies, except for oPt, where the association was only suggestive (p=0.14).(Table 5). The odds ranged from aOR=1.42 [95% CI 1.09-1.84] in the RRS-COMBAT sample to aOR=3.32 [95% CI 2.45- 4.5] in the Indashyikirwa sample. In the pooled analysis of all five studies, having depressive symptoms doubled the odds for physical IPV perpetration among men aOR=2.13 [95% CI 1.58-2.87]. In the three studies in which we asked about NPSV perpetration, the association between men's depressive symptomatology and NPSV perpetration was significant across the two South African studies (SSCF aOR=1.93 [95%CI 1.41- 2.64] and the Sonke Change Trial aOR=3.01[95%CI 2.5-3.61), but not the RRS-COMBAT study in Ghana. In the pooled analysis (3 studies), having depressive symptoms increased the odds for non-partner sexual violence perpetration more than one half (aOR=1.62 [95%CI 0.97- 2.71]), but this was not significant (p=0.06).

In the three studies where PTSD was measured, there were significant associations between men's PTSD symptomatology and past year physical IPV perpetration. Across individual studies (SSCF Sonke Change Trial and RRS-COMBAT) the adjusted association for PTSD symptomatology and past year IPV perpetration ranged from aOR=1.79 [95%CI 1.15-2.79] in SSCF to aOR=2.05[95%CI 1.14-4.0] in RRS-COMBAT (Table 5). In the pooled analysis among men who reported PTSD, the odds of perpetrating past year physical IPV were increased almost two-fold (aOR= 1.87 [95%CI 1.44-2.43]), compared to those who did not report PTSD symptoms in the past week. Post-traumatic Stress Disorder (PTSD) symptomatology was also significantly associated with NPSV perpetration across all three studies, with adjusted associations ranging from aOR=1.70 [95% CI 1.1-2.62] in Stepping Stones and Creating Futures Trial to aOR=3.08 [95%CI 2.13-4.45]. In the pooled analysis, the odds of perpetrating past year non-partner sexual violence were increased more than two-fold among men who reported PTSD aOR= 2.13 [95%CI 1.49-3.05] compared to those who did not report PTSD symptoms.

For peer review only - http://bmjopen.bmj.com/site/about/guidelines.xhtml

Table 5: Association between depression and PTSD and perpetration Physical IPV and Non-partner sexual violence†

			Past year physical IPV perpetration			Past year NPSV perpetration		
		**All (N)**	**n(%)**	**aOR(95% CI)**	**Study weight**	**n(%)**	**aOR(95% CI)**	**Study weight**
	i)**Depression**							
SSCF-SA	No	356	149(41.8)	Ref		113(31.7)	Ref	
	Yes	313	188(60.1)	2.14(1.57 , 2.91)	9.58	148(47.3)	1.93(1.41 , 2.64)	13.31
Sonke-SA	No	1695	534(31.5)	Ref		462(27.3)	Ref	
	Yes	698	416(59.6)	3.22(2.68 , 3.87)	34.17	371(53.0)	3.01(2.51 , 3.62)	47.42
RRS COMBAT-Ghana	No	1354	145(10.7)	Ref		139(10.3)	Ref	
	Yes	619	90(14.5)	1.56(1.17 , 2.070	28.27	57(9.2)	0.89(0.64 , 1.23)	39.26
Indashyikirwa-Rwanda	No	1357	271(20.0)	Ref		n/m	n/m	n/m
	Yes	289	131(45.3)	3.45(2.64 , 4.52)	23.57	n/m	n/m	n/m
oPt-Palestine	No	198	41(20.7)	Ref		n/m	n/m	n/m
	Yes	110	32(29.1)	1.46(0.84 , 2.52)	4.41	n/m	n/m	n/m
**Overall**	**No**	**4960**	**1140(23.0)**	**Ref**		**714(21.0)**	Ref	
	**Yes**	**2029**	**857(42.2)**	**2.13(1.58 , 2.87)**	**100**	**576(35.3)**	**1.62(0.97 , 2.71)**	**100**
	**ii) PTSD**							
SSCF-SA	No	572	276(48.2)	**Ref**		212(37.1)	**Ref**	
	yes	96	60(62.5)	1.79(1.15 , 2.79)	13.46	48(50.0)	1.70(1.10 , 2.62)	13.46
Sonke-SA	No	2204	833(37.8)	**Ref**		727(33.0)	**Ref**	
	Yes	126	76(60.3)	2.51(1.74 , 3.62)	46.8	76(60.3)	3.08(2.13 , 4.45)	46.8
RRS COMBAT- Ghana	No	1902	220(11.6)	**Ref**		180(9.5)	**Ref**	
	Yes	71	15(21.1)	2.05(1.14 , 3.68)	39.75	16(22.5)	2.78(1.56 , 4.96)	39.75
**Overall**	**No**	**4678**	**1329(28.4)**	**Ref**		**1119(23.9)**	**Ref**	
	**Yes**	**293**	**151(51.5)**	**1.87(1.44 , 2.43)**	**100**	**140(47.8)**	**2.13(1.49 , 3.05)**	**100**

†All models adjusted for participants age.

For peer review only - http://bmjopen.bmj.com/site/about/guidelines.xhtml

Association between violence experience and poor mental health

Among women across all studies, experiencing IPV in the past 12 months was significantly associated with reported depressive symptomatology with adjusted associations ranging from aOR= 2.13 [95% CI 1.54-2.92] in SSCF to aOR= 3.12 [95% CI 2.41-4.04] in RRS-COMBAT. In the pooled analysis, women who experienced IPV in the past 12 months, compared to those who had not, had a two and a half fold increased odds of experiencing depressive symptoms than those who did not aOR= 2.53 [95% CI 2.18-2.94].

Similarly, among women who had experienced NPSV in the past 12 months there were significant associations with depressive symptoms, with the adjusted associations ranging from aOR= 2.64 [95% CI 1.90-3.66] in SSCF Trial to aOR=2.54 [95% CI 1.47-4.42] in RRS-COMBAT. In the pooled analysis, women who experienced NPSV in the past 12 months had a more than two and a half fold increased odds of experiencing depressive symptoms than those who did not aOR= 2.65 [95% CI 2.02-3-46]. (See Table 6).

For peer review only - http://bmjopen.bmj.com/site/about/guidelines.xhtml

Table 6: **Association between IPV and NPSV and experience and depression among women†**

			Depression		
		All(N)	n(%)	aOR(95%CI)	Study weight
**Physical IPV experience**					
SSCF-SA	No	274	94(34.3)	Ref	
	yes	403	212(52.6)	2.11(1.54 , 2.90)	14.77
RRSCombat	No	1587	520(32.8)	Ref	
	Yes	290	175(60.3)	3.2(2.47 , 4.15)	40.94
Indashyikirwa-Rwanda	No	1031	193(18.7)	Ref	
	Yes	629	236(37.5)	2.69(2.14 , 3.37)	36.21
oPt-Palestine	No	277	134(48.4)	Ref	
	Yes	94	67(71.3)	2.83(1.7 , 4.71)	8.09
**All**	**No**	**3169**	**941(29.7)**	**Ref**	
	**Yes**	**1416**	**690(48.7)**	**2.53(2.18 , 2.94)**	**100**
**NPSV experience**					
SSCF-SA	No	449	167(37.2)	**Ref**	
	Yes	228	139(61.0)	2.64(1.9 , 3.67)	26.51
RRS-Combat	No	1823	663(36.4)	**Ref**	
	Yes	54	32(59.3)	2.82(1.62 , 4.91)	73.49
**All**	**No**	**2272**	**830(36.5)**	**Ref**	
	**Yes**	**282**	**171(60.6)**	**2.65(2.02 , 3.46)**	**100**

†All models adjusted for alcohol use and woman's age, except oPt, where model adjusted for age only.

Discussion

VAWG, and poor mental health are complex and overlapping problems, the results from this pooled analysis of data from from men and women participating in five IPV prevention studies across Africa and in the Middle East showed evidence of a clear association between poor mental health and men's perpetration of both physical intimate and non-partner sexual violence. Among women, we also found consistent associations between experiencing IPV and NPSV and reports of poor mental health outcomes.

There was a consistent positive association between men's depressive symptomatology and their perpetration of IPV in the pooled analysis, and in four out of the five country level analyses. These findings are similar to those of the UN Multi-country study which showed that men's depression was associated with physical and/or sexual IPV perpetration in three sites across Asia and the Pacific (Bangladesh, Cambodia, and China) (17). There are a few potential reasons why men's depressive symptoms may be associated with their perpetration of IPV. It may be that men who are depressed engage in more drinking behaviour, which in turn increases their risk of IPV/NPSV perpetration. This is in keeping with other

For peer review only - http://bmjopen.bmj.com/site/about/guidelines.xhtml

research that found poor mental health and alcohol are co-morbid and this increased men's risk of violence perpetration (18), as well as research from multiple low to middle income settings which indicate that men's drinking place women at increased risk of IPV and NPSV perpetration(27). It may also be that men who are depressed feel that they are unable to achieve traditional expectations placed on them, such as economic provision, or having stable employment, and in turn use seek to establish identity and power by controlling their partners. Previous research in informal settlements found that among men in contexts of poverty, unemployment and social marginalisation, controlling female partners has been used to consolidate social relationships, establish identity, power and respect (28).

The reason for the lack of association between depressive symptoms and men's IPV perpetration in oPt is unclear. Previous work has found that exposure to political violence places men at risk for perpetration of IPV(29), and this may mask other associations. Further work is required to understand the interrelationships between exposure to political violence (not measured here), men's mental health, and men's perpetration of IPV.

In this study, the association between men's depressive symptoms and perpetration of NPSV was significant in two of the three studies where NPSV was measured (SSCF and Sonke Change Trials), but not in RRS-COMBAT, the latter finding may be due to small number of men reporting depressive symptoms and non-partner sexual violence perpetration in the RRS-COMBAT intervention. Nonetheless, men who reported depressive symptoms did have an overall increased odds of perpetrating NPSV. An analysis of associations between past year NPSV perpetration and depression of men in a population based sample from Bougainville, Papua New Guinea in contrast did not show associations (30).

We found consistent positive associations between men's reports of PTSD symptoms and perpetration of IPV in 4 of the 5 studies and the pooled analysis, and for men's perpetration of NPSV, where this was significant in the pooled analysis and in all three of the individual studies (SSCF, Sonke and RRS-COMBAT) where NPSV was examined. There remains very little research on the association between PTSD and IPV and NPSV in general population samples. The evidence that there is primarily comes from largely military/offender populations (12, 13), and the analyses of these is complicated by the high rates of traumatic experiences that military staff may have experienced and other forms of violence in offender populations. The study of men in Bougainville, Papua New Guinea with very high population prevalence of PTSD and depression only found associations between past year depression and IPV perpetration, not with PTSD ((30). However, findings from our study extend understanding of PTSD as a risk factor for IPV in general populations by showing that PTSD doubles the risk for NPSV perpetration. This advances the

For peer review only - http://bmjopen.bmj.com/site/about/guidelines.xhtml

knowledge base, particularly in LMIC country settings, where non-partner sexual violence, particularly in Sub-Saharan African countries are prevalent.

The associations between women's experience of IPV and NPSV and depressive and PTSD symptoms, reflects the broader literature base (10). It also extends the knowledge base to include associations between NPSV experience among women and subsequent experience of PTSD. In addition, PTSD can be complex and often overlaps with depression among women. Therefore, disentangling the dimensions of PTSD and depression remains important to inform prevention programming and health service delivery among women. Prevention interventions need to address the mental health needs of women in prevention and response to IPV and NPSV. (31).

Strengths and Limitations

This study's key strengths is the pooled analysis of data from multiple settings and interventions across low to middle income setting in the global South, using comparable measures of men's IPV and NPSV perpetration, and women's experience of IPV and NPSV, and key covariates such as alcohol use. This addresses the limitation of many previous studies, which tend to focus on IPV only, and use differing measures of drivers and outcomes examining the associations between IPV and NPSV and mental health. The study also adds to the scant evidence base in the global South, where the co-occurring problems of poor mental health and VAWG are prevalent. Furthermore, it highlights the complex and overlapping nature of alcohol, poor mental health as drivers and outcomes of VAWG, often against the backdrop of other structural drivers, such as poverty, food insecurity and unemployment. However, we also recognize limitations in the research. Previous research suggests that there is likely a bi-directional relationship between recent IPV and poor mental health particularly for women (10, 32). However, the cross-sectional nature of the current data limits our ability to draw any conclusions about the temporal relationships, and necessitates future longitudinal analysis. Only two of the five studies were population-based and other studies were limited in generalizability, as they were based on populations recruited for the purpose of impact evaluation. Despite the current limitations, the study confirms that poor mental health are important drivers of men's use of violence, and women experiencing IPV and NPSV often experience poor mental health in LIMIC settings.

Conclusion

Our analysis has shown a consistent pattern of depression and PTSD symptoms as drivers of men's perpetration of IPV and NPSV, and increased depression and PTSD symptoms as outcomes among women who are in relationships characterised by IPV and NPSV. Interventions to address VAWG should more

For peer review only - http://bmjopen.bmj.com/site/about/guidelines.xhtml

actively consider addressing poor mental health as drivers, and address poor mental health outcomes among women experiencing IPV and NPSV in LMIC settings in prevention and treatment programmes.

Acknowledgements

The authors wish to thank the research teams, programme staff, and participants from all studies included in this pooled analysis.

Contributorship statement

LR conceptualized the analysis with AG and RJ. EC led the statistical analysis. AG was a PI on the Stepping Stones and Creating Futures study in South Africa, EC and DOA, and AAL were Co- PIs on the RRS-COMBAT study in Ghana, KD was a PI on the Indashyikirwa study in Rwanda, and MM is a VAWG and mental health researcher, who contributed to the overall drafting of the manuscript. RJ was the Director of the overall What Works to Prevent Violence Against Women and Girls Global programme. LR led the drafting of the manuscript; all authors contributed to comments and revisions.

Funding

All studies presented and this pooled analysis of the data were funded through the What Works to Prevent Violence? A Global Programme on Violence Against Women and Girls (VAWG) funded by the UK Government's Department for International Development (DFID), Grant number: PO 6254. However, the views expressed do not necessarily reflect the department's official policies and the funders had no role in study design; collection, management, analysis, and interpretation of data; writing of the report; and the decision to submit the paper for publication. Funding was managed by the South African Medical Research Council. Time drafting this manuscript was funded by the South African Medical Research Council.

Competing Interest

There are no potential conflicts of interest to declare.

Data sharing

De- identified individual participant data for Stepping Stones and Creating Futures (South Africa), Sonke Change trial (South Africa), and Evaluation of the RRS-COMBAT intervention (Ghana) and oPt

For peer review only - http://bmjopen.bmj.com/site/about/guidelines.xhtml

intervention , are available to anyone who wishes to access the data for any purpose at https://medat.samrc.ac.za/index.php/catalog/WW . De- identified individual participant data from the Indashyikirwa couples surveys (Rwanda) are available from the Principal Investigator of the study, Dr Kristin Dunkle: kristin.dunkle@mrc.ac.za , but may require permission from the Rwandan Ministry of Gender and Family Promotion (MIGEPROF) before transfer.

ORCID iD

Leane Ramsoomar: http://orcid.org/0000-0003-1934-579X

Andrew Gibbs: http://orcid.org/0000-0003-2812-5377

Esnat Chirwa: http://orcid.org/ 0000-0003-0471-4978

Kristin Dunkle: http://orcid.org/0000-0003-3071-5544

Rachel Jewkes http://orcid.org/0000-0002-4330-6267

Mercilene Machisa https://orcid.org/0000-0001-7275-1100

Deda Ogum-Alangea http://orcid.org/0000-0001-7496-8514

Adolphina Addo-Lartey https://orcid.org/0000-0001-8852-7189

Ethical issues

Ethical clearance for all studies was obtained prior to the studies commencing. For the Stepping Stones and Creating Futures Trial, clearance was obtained from the South African Medical Research Council's Ethics Committee (EC006-2/2015) and the University of KwaZulu-Natal's Biomedical Research Ethics Committee (BFC043/15). For the Sonke Change Trial, ethical clearance was obtained from the University of Witwatersrand's Ethics Committee (M150443). For the Indashyikirwa couple's intervention in Rwanda, ethical approval was obtained from the Rwandan National Ethics Committee (340/RNEC/2015) and the South Africa Medical Research Council Ethics Committee (EC033-10/2015). A required research permit was obtained from the National Institute of Statistics Rwanda (0738/2015/10/NISR). For the RRS-COMBAT intervention in Ghana, the Noguchi Memorial Institute for Medical Research, University of Ghana ((# 006/15–16) and the South African Medical Research Council's Ethics Committee (EC031-9/2015) granted ethical clearance for the study. Ethical clearance for the oPt study was granted by the South African Medical Research Council's ethics committee (EC014-5/2016). All studies followed ethical and safety guidelines for research on violence against women. All participants provided written informed

For peer review only - http://bmjopen.bmj.com/site/about/guidelines.xhtml

consent before participation. Further information on the studies ethics is available in the study sources referenced in Table 1.

For peer review only

For peer review only - http://bmjopen.bmj.com/site/about/guidelines.xhtml

References

1. World Health Organization. Violence against women prevalence estimates, 2018: Global, regional and national prevalence estimates for intimate partner violence against women and global and regional prevalence estimates for non-partner sexual violence against women. 2021.
2. Ellsberg M, Jansen HA, Heise L, Watts CH, Garcia-Moreno C. Intimate partner violence and women's physical and mental health in the WHO multi-country study on women's health and domestic violence: an observational study. The lancet. 2008;371(9619):1165-72.
3. Gibbs A, Dunkle K, Ramsoomar L, Willan S, Jama Shai N, Chatterji S, et al. New learnings on drivers of men's physical and/or sexual violence against their female partners, and women's experiences of this, and the implications for prevention interventions. Global health action. 2020;13(1):1739845.
4. Abramsky T, Watts CH, Garcia-Moreno C, Devries K, Kiss L, Ellsberg M, et al. What factors are associated with recent intimate partner violence? Findings from the WHO multi-country study on women's health and domestic violence. BMC public health. 2011;11(1):109.
5. Ellsberg M, Ovince J, Murphy M, Blackwell A, Reddy D, Stennes J, et al. No safe place: Prevalence and correlates of violence against conflict-affected women and girls in South Sudan. PLoS one. 2020;15(10):e0237965.
6. Stöckl H, Sardinha L, Maheu-Giroux M, Meyer SR, García-Moreno C. Physical, sexual and psychological intimate partner violence and non-partner sexual violence against women and girls: a systematic review protocol for producing global, regional and country estimates. BMJ open. 2021;11(8):e045574.
7. Machisa MT, Christofides N, Jewkes R. Mental ill health in structural pathways to women's experiences of intimate partner violence. PloS one. 2017;12(4):e0175240.
8. Machisa MT, Christofides N, Jewkes R. Structural pathways between child abuse, poor mental health outcomes and male-perpetrated intimate partner violence (IPV). PloS one. 2016;11(3):e0150986.
9. Lagdon S, Armour C, Stringer M. Adult experience of mental health outcomes as a result of intimate partner violence victimisation: a systematic review. European journal of psychotraumatology. 2014;5(1):24794.
10. Bacchus LJ, Ranganathan M, Watts C, Devries K. Recent intimate partner violence against women and health: a systematic review and meta-analysis of cohort studies. BMJ open. 2018;8(7):e019995.
11. Devries KM, Mak JY, Bacchus LJ, Child JC, Falder G, Petzold M, et al. Intimate partner violence and incident depressive symptoms and suicide attempts: a systematic review of longitudinal studies. PLoS medicine. 2013;10(5):e1001439.
12. Bell KM, Orcutt HK. Posttraumatic stress disorder and male-perpetrated intimate partner violence. JAmA. 2009;302(5):562-4.
13. Orcutt HK, King LA, King DW. Male-perpetrated violence among Vietnam veteran couples: relationships with veteran's early life characteristics, trauma history, and PTSD symptomatology. Journal of traumatic stress. 2003;16(4):381-90.
14. Trevillion K, Williamson E, Thandi G, Borschmann R, Oram S, Howard LM. A systematic review of mental disorders and perpetration of domestic violence among military populations. Social psychiatry and psychiatric epidemiology. 2015;50(9):1329-46.
15. Jordan BK, Marmar CR, Fairbank JA, Schlenger WE, Kulka RA, Hough RL, et al. Problems in families of male Vietnam veterans with posttraumatic stress disorder. Journal of consulting and clinical psychology. 1992;60(6):916.

For peer review only - http://bmjopen.bmj.com/site/about/guidelines.xhtml

16. King LA, King DW. Male-perpetrated domestic violence: Testing a series of multifactorial family models. Violence Against Women and Family Violence: Developments in Research, Practice, and Policy. 2004.
17. Fulu E, Jewkes R, Roselli T, Garcia-Moreno C. Prevalence of and factors associated with male perpetration of intimate partner violence: findings from the UN Multi-country Cross-sectional Study on Men and Violence in Asia and the Pacific. The lancet global health. 2013;1(4):e187-e207.
18. Machisa MT. The relationships between poor mental health, child abuse, women's experiences and male perpetration of intimate partner violence in Gauteng province of South Africa. Johannesburg: University of the Witwatersrand; 2019.
19. O'Donnell ML, Creamer M, Pattison P. Posttraumatic stress disorder and depression following trauma: understanding comorbidity. American Journal of Psychiatry. 2004;161(8):1390-6.
20. Hatcher AM, Gibbs A, Jewkes R, McBride R-S, Peacock D, Christofides N. Effect of childhood poverty and trauma on adult depressive symptoms among young men in peri-urban South African settlements. Journal of Adolescent Health. 2019;64(1):79-85.
21. Radloff LS. A self-report depression scale for research in the general population. Applied psychol Measurements. 1977;1:385-401.
22. Blodgett JM, Lachance CC, Stubbs B, Co M, Wu Y-T, Prina M, et al. A systematic review of the latent structure of the Center for Epidemiologic Studies Depression Scale (CES-D) amongst adolescents. BMC psychiatry. 2021;21(1):1-8.
23. Mollica RF, Caspi-Yavin Y, Bollini P, Truong T, Tor S, Lavelle J. The Harvard Trauma Questionnaire: validating a cross-cultural instrument for measuring torture, trauma, and posttraumatic stress disorder in Indochinese refugees. Journal of nervous and mental disease. 1992.
24. García-Moreno C, Jansen HA, Ellsberg M, Heise L, Watts C. WHO multi-country study on women's health and domestic violence against women: World Health Organization; 2005.
25. Jewkes R, Dunkle K, Koss MP, Levin JB, Nduna M, Jama N, et al. Rape perpetration by young, rural South African men: Prevalence, patterns and risk factors. Social science & medicine. 2006;63(11):2949-61.
26. Jewkes R, Fulu E, Tabassam Naved R, Chirwa E, Dunkle K, Haardörfer R, et al. Women's and men's reports of past-year prevalence of intimate partner violence and rape and women's risk factors for intimate partner violence: A multicountry cross-sectional study in Asia and the Pacific. PLoS medicine. 2017;14(9):e1002381.
27. Ramsoomar L, Gibbs A, Chirwa ED, Dunkle K, Jewkes R. Pooled analysis of the association between alcohol use and violence against women: evidence from four violence prevention studies in Africa. BMJ open. 2021;11(7):e049282.
28. Gibbs A, Govender K, Jewkes R. An exploratory analysis of factors associated with depression in a vulnerable group of young people living in informal settlements in South Africa. Global public health. 2018;13(7):788-803.
29. Riggs DS, Caulfield MB, Street AE. Risk for domestic violence: Factors associated with perpetration and victimization. Journal of clinical psychology. 2000;56(10):1289-316.
30. Jewkes R, Jama-Shai N, Sikweyiya Y. Enduring impact of conflict on mental health and gender-based violence perpetration in Bougainville, Papua New Guinea: a cross-sectional study. Plos one. 2017;12(10):e0186062.
31. Dixon LB, Holoshitz Y, Nossel I. Treatment engagement of individuals experiencing mental illness: review and update. World Psychiatry. 2016;15(1):13-20.
32. Devries KM, Child JC, Bacchus LJ, Mak J, Falder G, Graham K, et al. Intimate partner violence victimization and alcohol consumption in women: A systematic review and meta-analysis. Addiction. 2014;109(3):379-91.

For peer review only - http://bmjopen.bmj.com/site/about/guidelines.xhtml

For peer review only

For peer review only - http://bmjopen.bmj.com/site/about/guidelines.xhtml

BMJ Open

Pooled Analysis of the Association Between Mental Health and Violence Against Women: Evidence from five settings in the global South

Journal:	*BMJ Open*
Manuscript ID	bmjopen-2022-063730.R1
Article Type:	Original research
Date Submitted by the Author:	28-Oct-2022
Complete List of Authors:	Ramsoomar, Leane; South African Medical Research Council, Gender and Health Research Unit; University of Pretoria School of Health Systems and Public Health, School of Health Systems and Public Health<br>Gibbs, Andrew; University of Exeter School of Psychology, Gender and Health Research Unit; South African Medical Research Council, Gender and Health Research Unit<br>Chirwa, Esnat; South African Medical Research Council,<br>Machisa, Mercilene T.; South African Med Res Council, Gender and Health Research Unit<br>Alangea, Deda; University of Ghana School of Public Health, Public Health<br>Addo-Lartey, Adolphina Addoley ; University of Ghana,<br>Dunkle, Kristin ; South African Medical Research Council,<br>Jewkes, Rachel; South African Medical Research Council, Gender and Health Division ; University of the Witwatersrand Johannesburg School of Public Health, School of Public Health
**Primary Subject Heading**:	Public health
Secondary Subject Heading:	Global health
Keywords:	MENTAL HEALTH, Depression & mood disorders < PSYCHIATRY, PUBLIC HEALTH, Substance misuse < PSYCHIATRY, Public health < INFECTIOUS DISEASES, Anxiety disorders < PSYCHIATRY

SCHOLARONE™
Manuscripts

BMJ

I, the Submitting Author has the right to grant and does grant on behalf of all authors of the Work (as defined in the below author licence), an exclusive licence and/or a non-exclusive licence for contributions from authors who are: i) UK Crown employees; ii) where BMJ has agreed a CC-BY licence shall apply, and/or iii) in accordance with the terms applicable for US Federal Government officers or employees acting as part of their official duties; on a worldwide, perpetual, irrevocable, royalty-free basis to BMJ Publishing Group Ltd ("BMJ") its licensees and where the relevant Journal is co-owned by BMJ to the co-owners of the Journal, to publish the Work in this journal and any other BMJ products and to exploit all rights, as set out in our licence.

The Submitting Author accepts and understands that any supply made under these terms is made by BMJ to the Submitting Author unless you are acting as an employee on behalf of your employer or a postgraduate student of an affiliated institution which is paying any applicable article publishing charge ("APC") for Open Access articles. Where the Submitting Author wishes to make the Work available on an Open Access basis (and intends to pay the relevant APC), the terms of reuse of such Open Access shall be governed by a Creative Commons licence – details of these licences and which Creative Commons licence will apply to this Work are set out in our licence referred to above.

Other than as permitted in any relevant BMJ Author's Self Archiving Policies, I confirm this Work has not been accepted for publication elsewhere, is not being considered for publication elsewhere and does not duplicate material already published. I confirm all authors consent to publication of this Work and authorise the granting of this licence.

For peer review only

Pooled Analysis of the Association Between Mental Health and Violence Against Women: Evidence from five settings in the global South

Leane Ramsoomar[1,2,3,] Andrew Gibbs[1,4], Esnat Chirwa[1,2], Mercilene Machisa[1,2], Deda Ogum Alangea[5], Adolphina Addo-Lartey[5], Kristin Dunkle[1], Rachel Jewkes[1,2]

1 Gender and Health Research Unit, South African Medical Research Council, Pretoria, South Africa

2 School of Public Health, University of the Witwatersrand, Johannesburg, South Africa

3 School of Health Systems and Public Health, University of the Pretoria, Gauteng, South Africa

4 Centre for Rural Health, School of Nursing and Public Health, University of KwaZulu-Natal, Durban, South Africa

5 School of Public Health, University of Ghana

Corresponding author:

Leane Ramsoomar [1,2,3] leane.ramsoomar@mrc.ac.za

[1] Gender and Health Research Unit, South African Medical Research Council, Pretoria, South Africa

[2] School of Public Health, University of the Witwatersrand, Johannesburg, South Africa

[3] School of Health Systems and Public Health, University of the Pretoria, Gauteng, South Africa

Abstract

Objectives: To describe associations between men's poor mental health (depressive and post-traumatic stress symptomatology) and their perpetration of Intimate Partner Violence (IPV) and non-partner sexual violence (NPSV), and women's mental health and their experiences of IPV and NPSV in five settings in the global South.

Design: A pooled analysis of data from baseline interviews with men and women participating in five Violence against Women and Girls (VAWG) prevention intervention evaluations.

Setting: Three Sub-Saharan African countries [South Africa (2), Ghana, and Rwanda], and one Middle Eastern country, the occupied Palestinian territories (oPt).

Participants: 7021 men and 4,525 women 18+ years old from a mix of self-selecting and randomly selected household surveys.

Main outcome measures: All studies measured depression symptomatology using the Centre for Epidemiological Studies-Depression (CESD), and the Harvard Trauma Scale for PTSD symptoms among

men and women. IPV and NPSV were measured using items from modified WHO Women's Health and Domestic Violence and UN Multi-country Study to assess perpetration among men, and experience among women.

Findings: Overall men's poor mental health was associated with increased odds of perpetrating physical IPV and NPSV. Specifically, men who had more depressive symptoms had increased odds of reporting IPV (aOR=2.13 [95%CI 1.58-2.87]) and NPSV perpetration (aOR= (1.62 95% CI 0.97 -2.71) compared to those with fewer symptoms. Men reporting PTSD had higher odds of reporting IPV (aOR= 1.87 [95% CI 1.44 -2.43]) and NPSV (aOR= 2.13 [95% CI 1.49 -3.05]) perpetration compared to those without PTSD. Women who had experienced IPV (aOR= 2.53 [95% CI 2.18 -2.94]) and NPSV (aOR= 2.65 [95% CI 2.02 -3.46]) had increased odds of experiencing depressive symptoms compared to those who had not.

Conclusions: Interventions aimed at preventing IPV and NPSV perpetration and experience must account for the mental health of men as a risk factor, and women's experience.

Article Summary

Strengths

- Synthesises comparable data across LMIC settings in the global South, thereby addressing the limited geographical scope of studies examining the association between mental health as a driver of men's violence perpetration against women, and as a consequence of violence experienced by women in the global South.
- Addresses both IPV and NPSV perpetration by men and experience by women using comparable measures.
- Highlights the need to integrate mental health focus into programmes aimed to prevent male to female intimate and non-intimate partner violence and interventions to address poor mental health outcomes among women experiencing IPV and NPSV in LMIC settings.

Limitations

- Only two of the five studies are population-based, limiting generalizability.
- All data are cross-sectional, limiting inference about causality and direction of effects.

For peer review only - http://bmjopen.bmj.com/site/about/guidelines.xhtml

Introduction

Violence against women and girls (VAWG), particularly, intimate partner violence (IPV) and non-partner sexual violence (NPSV) are significant global public health problems. Globally, about 35% of women report having experienced either physical and/or sexual intimate partner violence or non-partner sexual violence in their lifetime (1). A large body of research has emerged identifying drivers of men's perpetration of physical and or sexual violence against their female partners, and women's experience of this, in diverse contexts and populations. Drivers of violence perpetration and experience include poverty, gender inequalities, normalization of violence, substance use, exposure to abuse as a child, and poor communication in relationships and conflict skills, as well as living conflict-affected settings (2-5). Research has established associations between experience of IPV and a range of negative health outcomes, particularly mental health outcomes, HIV, low birthweight, termination of pregnancy, suicidal ideation and harmful alcohol use (1, 6).

Poor mental health is increasingly recognized as both an outcome and a driver of VAWG (7, 8). Among women, the associations between VAWG and negative health outcomes have been recognised for some time and poor mental health has largely been established as an outcome of IPV and NPSV experience (9). The WHO multi-country study on Women's Health and Domestic violence found significant associations between lifetime experiences of partner violence and self-reported poor health, including more emotional distress, suicidal thoughts and attempts among abused, compared with non-abused, women (2). A systematic review and meta-analysis of cohort studies found a strong association between recent IPV experience and depressive symptoms among women(10). Research has shown that poor mental health may also be a risk factor for women's IPV experience. For instance, Devries and colleagues found that women who experience depression may be more accepting of partners with traits that predispose them to violence (11), while other research found that women who experience depression may withdraw or display lethargy, which impacts their ability to seek help, or remove themselves from relationships (10). Furthermore, the systematic review of cohort studies (mentioned above), found evidence of a bi-directional relationship between depression and IPV experience, whereby women's depressive symptoms were associated with subsequent IPV experience(10).

Among men, research has found that poor mental health is associated with IPV perpetration, but the majority of this evidence has come largely from military or male offender samples from high income countries (12-14). There is, however, heterogeneity among samples and measures and definitions of IPV in previous work. Additionally this prior research has often focused on symptoms of PTSD (8, 13, 15, 16),

For peer review only

with evidence indicating strong associations between PTSD and IPV perpetration (13). Depression as a risk factor for male perpetration of violence against women has been less frequently studied and where examined, results have been mixed. For example, the cross-sectional population-based UN multi-country study in the Asia-Pacific region found that depression was associated with men's physical and sexual IPV perpetration in samples from four of six countries (17). However, evidence from a systematic review of population based studies found no association between depression and IPV perpetration (18). This may be due to limited numbers of population-based studies on men's violence perpetration, and the frequent omission of measures of men's mental health in studies of IPV perpetration. To our knowledge the association between men's poor mental health and their risk of NPSV perpetration has not been examined in LMICs.

Poor mental health often occurs against the backdrop of poverty, adverse childhood experiences, life traumatic events, low education and gender inequality and conflict and the wider context of generalized violence (3, 8, 19, 20). These factors operate either directly or indirectly to impact on childhood adverse events, poor mental health, substance use and relationship conflict, which in turn increase risk for IPV (3). Data from a population-based survey in South Africa found that experiencing adverse childhood events (physical, sexual and emotional abuse) increased the risk of men's poor mental health (PTSD, depression), harmful alcohol use, which in turn increased the likelihood of male-perpetrated IPV (8).

Although poor mental health has been recognized as both a driver and outcome of VAWG, the majority of studies are from high-income settings, focus largely on IPV only, and use differing measures of exposure and outcomes, which limits comparison across settings. There is much less research on these associations in low-middle income settings, where co-occurring public health challenges of VAWG, poor mental health and harmful alcohol use are prevalent. To strengthen the evidence base from low- and middle-income countries, and advance the current literature base on the role of poor mental health in both IPV and NPSV perpetration and victimisation, we undertook a pooled analysis of baseline data using comparable measurement methods from five IPV prevention studies conducted in three countries in Africa (South Africa – which had two studies - Ghana and Rwanda) and one conflict-afflicted country in the Middle East, the occupied Palestinian Territories (oPt) [West Bank and Gaza]. All studies report on recent (past 12 month) IPV and NPSV experience/perpetration, as opposed to lifetime exposure to IPV and NPSV. The paper aims to answer the following research questions (i) Is men's poor mental health

For peer review only - http://bmjopen.bmj.com/site/about/guidelines.xhtml

associated with IPV and NPSV perpetration ? (ii) Is women's experience of IPV and NPSV associated with poor mental health outcomes?.

Methods

The studies included in this pooled analysis were conducted under the UK-Aid funded What Works to Prevent Violence Against Women and Girls? Global Programme (What Works). The primary goal of What Works was to advance the evidence base on the prevalence and drivers of VAWG in the Global South, and determine the effectiveness of interventions to prevent VAWG. The current study used the baseline data from 7021 men and 4,525 women from five VAWG prevention studies in four countries (South Africa, Ghana, Rwanda, and occupied Palestinian Territories [West Bank and Gaza]) to assess the association between poor mental health (depression and PTSD symptomatology) and IPV and NPSV perpetration among men and the association between poor mental health and IPV and NPSV experience among women. These studies include the Stepping Stones and Creating Futures intervention (South Africa), the Sonke Change trial (South Africa), the Rural Response System (RRS-COMBAT) community intervention (Ghana), the Indashyikirwa couples intervention (Rwanda), and a population-based nationally representative survey in the occupied Palestinian Territory (oPt) [West Bank and Gaza]. Further information on the studies is available in the study sources referenced in Table 1.

Patient and public involvement

Patients and the public were not involved in the study design, implementation, or analysis presented in this study. However, the individual projects had different levels of participant and public involvement as part of the wider What Works research uptake strategy.

For peer review only - http://bmjopen.bmj.com/site/about/guidelines.xhtml

Table 1: **Datasets used for men and women included in the pooled analysis**

#	Study	Country	Study Design	# clusters	N (Men)	N (Women)	Sampling or recruitment strategy	Age
1	Evaluation of Stepping Stones and Creating Futures	South Africa	CRCT	34	674	677	Study volunteers	18-35
2	Evaluation of Sonke Change trial	South Africa	CRCT	18	2406	-	Household-based random sample survey	18-45
3	Evaluation of the RRS-COMBAT community intervention	Ghana	CRCT	40	1973	1877	Household-based random sample survey	18+ (men) 18-45 (women)
4	Indashyikirwa -- couples intervention	Rwanda	CRCT	28	1651	1660	Volunteer recruitment from savings and loan association groups	18-50
5	Evaluation of Innovative Media to End VAWG through Community Education and Outreach in the oPt	Occupied Palestinian Territories	Population-based survey	55	308	371	Population-based nationally representative survey	18+ (men) 18+ (women)

For peer review only

Measures

All study measures are presented in Table 2. We assessed socio-demographics for men and women, including, age, current marital status, relationship residence status, and education, employment in the past 3 months and past year, respectively. All studies except Indashyikirwa (couples) asked whether participants had worked in the past 3-months or the past year respectively; the two South African studies asked whether female participants had worked in the past 3-months or not. Income assessments in Indashyikirwa couples were not included in the present analyses, as they were tailored to the local context in which most participants engaged in subsistence agriculture.

Depression symptoms were measured using the previously validated Center for the Epidemiological Studies of Depression Short Form (CESD-10) (21, 22). PTSD symptoms were measured in three of the five studies (SSCF, Sonke Change trial, Ghana (men only), using the previously validated Harvard Trauma questionnaire (23), in settings where it was anticipated that the intervention would impact it. Men's current alcohol use was assessed by asking one question about alcohol use in the 12 months preceding the baseline data collection, while women were asked if they had seen their partner drunk, and how frequently they saw them drunk in the past 12 months. Violence perpetration (IPV and NPSV) and experience (IPV) were measured using items from the WHO Women's Health and Domestic Violence survey (24), modified appropriately to assess men's perpetration (17). The NPSV scale was first developed in South Africa (25) and subsequently refined and used extensively in the Asia-Pacific region (26). There was no question on NPSV perpetration in the Indashyikirwa couples or the oPt, because of concerns about the particular sensitivity of the questions in those contexts.

For peer review only - http://bmjopen.bmj.com/site/about/guidelines.xhtml

Table 2: **Key Measures**

CONSTRUCT	Indicator	Definition
**VIOLENCE AGAINST WOMEN**	Physical IPV perpetration	Five items were used to measure men's physical IPV perpetration including: In the last 12 months how many times (1) did you slap your current or previous girlfriend or wife or throw something at her which could hurt her? (2) have you pushed or shoved a current or previous girlfriend or wife? (3) have you hit a current or previous girlfriend or wife with a fist or with something else which could hurt her? (4) did you kick, drag, beat, choke or burn a previous or current girlfriend, partner or wife? (5) did you threaten to use or actually use a gun, knife or other weapon against a previous or current girlfriend, partner or wife? Responses: "Never", "Once" "A few times" or "Many times". Men who responded "Once" or more to one or more items were coded as perpetrating physical IPV. The items were developed during the WHO Women's Health and Domestic Violence survey, and UN Multi-country study on Men and Violence in Asia and the Pacific, modified to assess men's perpetration of physical and NPSV in the past year(17, 24).
	Physical IPV experience	Five items used to capture women's violence experience including: In the past 12 months, (1) how many times has a current or previous husband (or boyfriend) slapped you or thrown something at you which could hurt you? (2) pushed or shoved you? (3) hit you with a fist or with something else which could hurt you? (4) kicked, dragged, beaten, choked or burnt you? (5) threatened to use, or actually used, a gun, knife or other weapon against you?(24) Responses: "Never", "Once" "A few times" or "Many times". Women who responded "Once" or more to one or more items were coded as experiencing physical IPV.
	NPSV perpetration	Five items about NPSV perpetration, including: In the last 12 months how many times have you: (1) forced or persuaded a woman or girl who was not your girlfriend or wife at the time to have sex with you? (2) ever tried to force or persuade any woman or girl who was not your girlfriend or partner to have sex with you,

For peer review only - http://bmjopen.bmj.com/site/about/guidelines.xhtml

		but did not succeed? (3) ever had sex with a woman or girl who was not your girlfriend or wife when she was too drunk or drugged to stop you? (4) have you and other men ever had sex with a woman or girl at the same time who was not your girlfriend or wife when she did not agree to sex, or you forced her? (5) have you and other men ever had sex with a woman or girl at the same time who was not your girlfriend or wife when she was too drunk or drugged to stop you? Responses: Never", "Once" "A few times" or "Many times". Men who responded "Once" or more to one or more items were coded as perpetrating NPSV. The scale was first developed in South Africa and subsequently refined and used extensively in Asia-Pacific region(26)
	NPSV experience	Six items about NPSV perpetration, including: In the past 12 months. (1) How many times has any man who is NOT your boyfriend or husband forced or persuaded you to have sex against your will?... (2)tried to force you to have sex against your will and did not succeed?... (3) forced you to have sex against your will when you were too drunk or drugged to refuse?... (4) did two or more men force you to have sex with them at the same time against your will?... (5) did two or more men force you to have sex with them at the same time against your will when you were too drunk or drugged to refuse?... (6) was there an occasion when you agreed to have sex with one man and one or more others who you had not agreed to have sex with forced you to have sex with them as well? This was coded in the same way as for the men.
**ALCOHOL USE**	Current Alcohol Use (past 12 months)	Current alcohol use by men was measured by asking one question about alcohol use in the 12 months preceding the baseline data collection: (1) Have you drunk alcohol in the past 12 months?. Responses were either "Yes" or "No".
	Seen partner drunk	1 item assessing if women has seen their partner drunk in the past 12 months. Responses: "Yes" or "No"
	Frequency of seeing partner drunk	1 item assessing how often women had seen their partner drunk in the past 12 months. Responses were "every day or nearly every day" "Weekly" " Once a month" " Less than once a month" " Never" and recoded into: "never" "occasionally" "Frequently"

For peer review only - http://bmjopen.bmj.com/site/about/guidelines.xhtml

**MENTAL HEALTH**	Depression symptoms (men and women)	Three studies (SSCF, Sonke Change Trial and RRS-COMBAT) used the 20-item Centre for Epidemiological Studies Depression (CESD) scale used to measure depression symptoms. Items were framed around statements about feelings in the past week, such as: 'During the past week I felt fearful' with responses, 'none or rarely', 'some or a little', 'moderate amount of time', and 'most or all the time'. Items were summed, and ranged between 0-41, with higher scores indicating more depressive symptoms (Cronbach alpha ranged from 0.78-0.91 across studies). Three studies (Indashyikirwa and oPt) used the 10-item Centre for Epidemiological Studies Depression (CESD) short form scale to measure depression symptoms. Items were summed, and ranged between 0-21, with higher scores indicating more depressive symptoms.
	Post-Traumatic Stress Disorder (PTSD) symptoms (men and women).	Harvard Trauma Scale for PTSD, which reflected reactions to traumatic experiences in their lifetime experienced in the past week, such as: " In the past week have you had recurrent thoughts or memories of the most hurtful or terrifying events" or " . In the past week have you had recurrent nightmares". Responses were rated 0= not at all, to 3= extremely. Items were summed with higher scores indicating more PTSD symptoms. Cronbach alphas ranged from 0.91-0.94 across studies where PTSD was measured.

For peer review only - http://bmjopen.bmj.com/site/about/guidelines.xhtml

Data analysis

Descriptive statistics (frequencies and percentages) were used to summarize participants' socio-demographic characteristics within each study and in the pooled analysis. Within-study and pooled estimates considered any clustering with each study's sampling procedures. All pooled estimates were weighted according to the study sample size. We used forest plots, I-square and Cochran's Q statistics to assess the consistency of outcomes across the studies. The I-square values showed high heterogeneity in physical IPV as an outcome (80%, p-value<0.001 for men) and low heterogeneity for depression symptoms among women (14.8%, p-value=0.329). We used mixed-effects models to estimate overall effects and account for any heterogeneity across the studies due to methodological diversity. We fitted a one-stage Individual Patient Data (IPD) meta-analysis using mixed-effects logistic regression models to account for within-and between-study variances (heterogeneity) across studies for both men and women (17). We derived study-specific estimates and forest plots from a post-estimation model of the mixed-effects logistic regression model. Both the main and post-estimation models included participants' age as fixed effects. All data were analyzed using Stata Statistical Software: Release 17. College Station, TX: StataCorp LLC. StataCorp. 2019 and all models were adjusted for participants' age and alcohol use or partner alcohol use (for women), because of the association between age and IPV experience/perpetration, and co-morbidity between alcohol, VAWG and poor mental health found in previous research.

Results

Men

The mean age of men across all studies was 33.1 years old (SD=11.4). Forty-five percent of men were married while 45% were in a non-marital relationship. Overall, only 10% of men across all studies were not in a relationship; 61% of men indicated they were living with their partners, except in Rwanda (100%) couples and oPt (95%) where the majority were married. Almost a third of men (29%) reported not living together with their female partners. Most of the men had secondary school education or above (66%) and half (53%) the men in three studies were employed in the past 3 months (we did not ask about employment status in Rwanda and the oPt). (Table 3)

Women

The mean age of women across the four studies was 31.3 years old (SD=8.4). Over half (56%) of women were married, 38% were in a relationship, 75% were living with their partner or spouse and 19% were not living with their partner, and 6% were not in a relationship. Nearly one-half (48%) of the women had

For peer review only - http://bmjopen.bmj.com/site/about/guidelines.xhtml

completed secondary school education or above, and 53% reported working in the past 3 months, though this was not asked in Rwanda or oPt. Just over a third of women (32%) reported that their partner consumed alcohol in the past month. (See Table 3)

For peer review only

Table 3: **Socio-demographics, prevalence of alcohol consumption among men, reports of partner alcohol consumption among women in the included studies**

	Stepping Stones and Creating Futures Trial, South Africa	Sonke Change Trial, South Africa	RRS COMBAT, Ghana	Indashyikirwa - couples, Rwanda	oPt (Palestine)	All studies
	n(%) /mean(sd)	n(%)/mean(sd)	n(%) /mean(sd)	n(%) /mean(sd)	n(%) /mean(sd)	n(%) /mean(sd)
**MEN**	**(n=674)**	**(n=2406)**	**(n=1973)**	**(n=1651)**	**(n=317)**	**(n=7021)**
**Age**	23.8(3.6)	27.6(5.7)	39.3(14.9)	35.6(7.1)	43.9(13.8)	33.1(11.4)
**Current marital status**						
Married	22(3.3)	448(18.6)	1271(64.4)	1095(66.3)	301(95.0)	3137(44.7)
In a relationship	508(75.4)	1539(64.0)	533(27.0)	556(33.7)	0(0)	3136(44.7)
No relationship	144(21.4)	391(16.2)	169(8.6)		16(5.0)	720(10.3)
missing	0(0)	28(1.2)	0(0)			28(0.4)
**Relationship residence status**						
Living together	73(10.8)	932(38.7)	1314(66.6)	1651(100)	301(95.0)	4271(60.8)
Not living together	457(67.8)	1055(43.9)	490(24.8)		0(0)	2002(28.5)
No relationship	144(21.4)	391(16.2)	169(8.6)		16(5.0)	720(10.3)
missing	0(0)	28(1.2)	0(0)			28(0.4)
**Education**						
none	0(0)	0(0)	372(18.9)	265(16.1)	14(4.4)	651(9.3)
Primary school	77(11.4)	140(5.8)	334(16.9)	1088(65.9)	75(23.7)	1714(24.4)
Sec school or above	597(88.6)	2249(93.5)	1267(64.2)	298(18)	227(71.6)	4638(66.1)
missing	0(0)	17(0.7)	0(0)		1(0.3	18(0.3)
**Employed in past 3 months**	240(35.7)	1192(50)	1231(71.6)	n/m*	n/m	2663(52.7)
**Employed in past year**	156(23.2)	806(33.7)	1251(63.4)	n/m	n/m	2213(43.8)
**Drinks alcohol**	294(43.6)	948(39.4)	291(15.0)	246(15.0)	n/m	1779(27.0)

For peer review only - http://bmjopen.bmj.com/site/about/guidelines.xhtml

For peer review only

**WOMEN**	**(n=677)**	-	**(n=1877)**	**(n=1660)**	**(n=371)**	**(n=4585)**
**Age in yrs – mean(sd)**	23.9(3.6)		31.4(8.5)	32.7(6.6)	37.7(11.7)	31.3(8.4)
**Current marital status**						
Married	29(4.28)	-	1068(56.9)	1096(66)	365(98.4)	2558(55.8)
In a relationship	524(77.4)	-	664(35.4)	564(34)	0(0)	1752(38.2)
No relationship	124(18.3)	-	145(7.7)	0(0)	6(1.6)	275(6)
**Relationship residence status**						
Living together	113(16.7)	-	1303(69.4)	1660(100)	365(98.4)	3441(75.1)
Not living together	440(65.0)	-	429(22.9)	0(0)	0(0)	869(19)
No relationship	124(18.3)	-	145(7.7)	0(0)	6(1.6)	275(6)
**Education**						
None	0(0)	-	401(21.4)	288(17.4)	3(0.8)	692(15.1)
Primary school	56(8.3)	-	426(22.7)	1115(67.2)	120(32.4)	1717(37.5)
Sec school or above	621(91.7)	-	1050(55.9)	257(15.5)	248(66.8)	2176(47.5)
**Employed in past 3 months**	173(25.6)	-	1174(62.7)	n/m	n/m	1347(52.8)
**Employed in past year**	97(14.3)	-	1034(55.1)	n/m	n/m	1131(44.3)
**Seen Partner frequently drunk in past year**	357(52.7)		268(14.3)	742(44.7)	n/m	1367(32.4)
Never	320 (47.3)	-	1609 (85.7)	918 (55.3)	n/m	2847
Occasionally	220 (32.5)	-	115 (6.1)	471 (28.4)	n/m	806
Frequently	137 (20.2)	-	153 (8.2	271 (16.3)	n/m	561

* not measured

- Men only sample

For peer review only - http://bmjopen.bmj.com/site/about/guidelines.xhtml

IPV and NPSV perpetration, poor mental health and alcohol consumption among men

Among the 7,021 men across the studies, almost a third (29%) reported perpetrating past year intimate partner violence, with study-specific prevalence ranging from 12% (Ghana) to 50% (SSCF). Across the three studies where men were asked about perpetration of NPSV, 26% of men reported perpetrating past year NPSV, ranging from 10% in Ghana to 39% in SSCF. We did not ask about the perpetration of NPSV in Rwanda or oPt as we were advised by the local partner that these questions would be too sensitive for these socio-political contexts. . Overall, 29% of men reported depressive symptoms (range: 18% to 46%), and in the three studies where we asked about PTSD symptomatology (SSCF, Sonke Change trial and Ghana), 6% reported PTSD (range: 5-14.2%). In four studies where alcohol was asked about, 68% of men reported alcohol consumption in the past year (SSCF); 64% in the Sonke Change Trial; 44% in RRS-COMBAT, and 37% in Indashyikirwa. Alcohol consumption was not included in the questionnaire in the oPt. (See Table 4)

Women

IPV and NPSV experience, poor mental health, and reports of partner alcohol consumption

Among the 4,585 women across the studies, just over a third (31%) reported experiencing past year intimate partner violence, with study-specific prevalence ranging from 16% (Ghana) to 60% (SSCF). Across the two studies where NPSV was reported (SSCF and RRS-COMBAT), 11% of women reported experiencing past year NPSV, ranging from 3% in Ghana to 34% in SSCF. Overall, 36% of women reported depressive symptoms (range: 26% to 45%). Only women in SSCF were asked about PTSD symptomatology, and 21% reported such symptoms. (See Table 4)

For peer review only - http://bmjopen.bmj.com/site/about/guidelines.xhtml

Table 4: **Prevalence of Depression, PTSD symptomatology, Physical IPV and NPSV perpetration among men and experience among women in the studies included**

		Depression	PTSD	Physical IPV	NPSV
**MEN**					
	**N**	**n(%)**	**n(%)**	**n(%)**	**n(%)**
SSCF-SA	674	313(46.4)	96(14.2)	337(50.0)	261(38.7)
Sonke-SA	2406	700(29.1)	126(5.0)	952(39.6)	834(34.7)
RRS COMBAT-Ghana	1973	619(31.4)	71(3.60)	235(11.9)	196(9.9)
Indashyikirwa-Rwanda	1651	290(17.6)	n/m	402(24.4)	n/m*
oPt-Palestine	308	112(35.3)	n/m	73(23.0)	n/m
**Overall**	**7021**	**2034(29.0)**	**293(5.8)**	**1999(28.5)**	**1291(25.5)**
**WOMEN**					
	**N**	**Depression**	**PTSD**	**Physical IPV**	**NPSV**
SSCF-SA	677	306(45.2)	142(21.0)	403(59.5)	228(33.7)
RRS COMBAT-Ghana	1877	695(37.0)	*n/m	290(15.5)	54(2.88)
Indashyikirwa-Rwanda	1660	429(25.8)	n/m	629(37.9)	n/m
oPt-Palestine	371	201(54.2)	n/m	94(25.3)	n/m
**Overall**	**4585**	**1631(35.6)**	**142(21.0)**	**1416(30.9)**	**282(11.0)**

* not measured

For peer review only - http://bmjopen.bmj.com/site/about/guidelines.xhtml

Men

Association between poor mental health and men's violence perpetration

The association between men's depressive symptomatology and perpetration of past year IPV was significant across four studies, except for oPt, where the association was only suggestive (p=0.14).(Table 5). The odds ranged from aOR=1.56 [95% CI 1.17-2.07] in the RRS-COMBAT sample to aOR=3.45 [95% CI 2.64- 4.52] in the Indashyikirwa sample. In the pooled analysis of all five studies, having depressive symptoms increased the odds for physical IPV perpetration among men aOR=2.13 [95% CI 1.58-2.87]. In the three studies in which we asked about NPSV perpetration, the association between men's depressive symptomatology and NPSV perpetration was significant across the two South African studies (SSCF aOR=1.93 [95%CI 1.41- 2.64] and the Sonke Change Trial aOR=3.01[95%CI 2.51-3.62), but not the RRS-COMBAT study in Ghana. In the pooled analysis (3 studies), having depressive symptoms increased the odds for non-partner sexual violence perpetration more than one half (aOR=1.62 [95%CI 0.97- 2.71]), but this was not significant (p=0.06).

In the three studies where PTSD was measured, there were significant associations between men's PTSD symptomatology and past year physical IPV perpetration. Across individual studies (SSCF, Sonke Change Trial and RRS-COMBAT) the adjusted association for PTSD symptomatology and past year IPV perpetration ranged from aOR=1.79 [95%CI 1.15-2.79] in SSCF to aOR=2.51[95%CI 1.74-3.62] in the Sonke Change Trial (Table 5). In the pooled analysis among men who reported PTSD, the odds of perpetrating past year physical IPV were increased almost two-fold (aOR= 1.87 [95%CI 1.44-2.43]), compared to those who did not report PTSD symptoms. Post-traumatic Stress Disorder (PTSD) symptomatology was also significantly associated with NPSV perpetration across all three studies, with adjusted associations ranging from aOR=1.70 [95% CI 1.10-2.62] in the Stepping Stones and Creating Futures Trial to aOR=3.08 [95%CI 2.13-4.45] in the Sonke Change Trial. In the pooled analysis, the odds of perpetrating past year non-partner sexual violence were increased more than two-fold among men who reported PTSD aOR= 2.13 [95%CI 1.49-3.05] compared to those who did not report PTSD symptoms.

For peer review only - http://bmjopen.bmj.com/site/about/guidelines.xhtml

Table 5: Association between depression and PTSD symptoms and perpetration Physical IPV and Non-partner sexual violence

			Past year physical IPV perpetration			Past year NPSV perpetration		
		All (N)	n(%)	aOR(95% CI)†	Study weight	n(%)	aOR(95% CI)	Study weight
	**Depression**							
SSCF-SA	No	356	149(41.8)	Ref		113(31.7)	Ref	
	Yes	313	188(60.1)	2.14(1.57 , 2.91)	9.58	148(47.3)	1.93(1.41 , 2.64)	13.31
Sonke-SA	No	1695	534(31.5)	Ref		462(27.3)	Ref	
	Yes	698	416(59.6)	3.22(2.68 , 3.87)	34.17	371(53.0)	3.01(2.51 , 3.62)	47.42
RRS COMBAT-Ghana	No	1354	145(10.7)	Ref		139(10.3)	Ref	
	Yes	619	90(14.5)	1.56(1.17 , 2.07)	28.27	57(9.2)	0.89(0.64 , 1.23)	39.26
Indashyikirwa-Rwanda	No	1357	271(20.0)	Ref		n/m	n/m	n/m
	Yes	289	131(45.3)	3.45(2.64 , 4.52)	23.57	n/m	n/m	n/m
oPt-Palestine	No	198	41(20.7)	Ref		n/m	n/m	n/m
	Yes	110	32(29.1)	1.46(0.84 , 2.52)	4.41	n/m	n/m	n/m
**Overall**	No	4960	1140(23.0)	Ref		714(21.0)	Ref	
	Yes	2029	857(42.2)	2.13(1.58 , 2.87)	100	576(35.3)	1.62(0.97 , 2.71)	100
	**PTSD**							
SSCF-SA	No	572	276(48.2)	Ref		212(37.1)	Ref	
	yes	96	60(62.5)	1.79(1.15 , 2.79)	13.46	48(50.0)	1.70(1.10 , 2.62)	13.46
Sonke-SA	No	2204	833(37.8)	Ref		727(33.0)	Ref	
	Yes	126	76(60.3)	2.51(1.74 , 3.62)	46.8	76(60.3)	3.08(2.13 , 4.45)	46.8
RRS COMBAT- Ghana	No	1902	220(11.6)	Ref		180(9.5)	Ref	
	Yes	71	15(21.1)	2.05(1.14 , 3.68)	39.75	16(22.5)	2.78(1.56 , 4.96)	39.75
**Overall**	No	4678	1329(28.4)	Ref		1119(23.9)	Ref	
	Yes	293	151(51.5)	1.87(1.44 , 2.43)	100	140(47.8)	2.13(1.49 , 3.05)	100

† All models adjusted for participants age only

For peer review only - http://bmjopen.bmj.com/site/about/guidelines.xhtml

Among women across all studies, experiencing IPV in the past 12 months was significantly associated with reported depressive symptomatology with adjusted associations ranging from aOR= 2.11 [95% CI 1.54-2.90] in SSCF to aOR= 3.21 [95% CI 2.47-4.15] in RRS-COMBAT. In the pooled analysis, women who experienced IPV in the past 12 months, compared to those who had not, had a two and a half fold increased odds of experiencing depressive symptoms than those who did not aOR= 2.53 [95% CI 2.18-2.94].

Similarly, among women who had experienced NPSV in the past 12 months there were significant associations with depressive symptoms, with the adjusted associations ranging from aOR=2.64 [95% CI 1.90-3.67] in the SSCF to aOR= 2.82 [95% CI 1.62-4.91] in the RRS-COMBAT to. In the pooled analysis, women who experienced NPSV in the past 12 months had a more than two and a half fold increased odds of experiencing depressive symptoms than those who did not aOR= 2.65 [95% CI 2.02-3-46]. (See Table 6).

For peer review only - http://bmjopen.bmj.com/site/about/guidelines.xhtml

Table 6: **Association between IPV and NPSV and experience and depressive symptoms among women**

			Depression		
		All(N)	n(%)	aOR(95%CI) †	Study weight
**Physical IPV experience**					
SSCF-SA	No	274	94(34.3)	Ref	
	yes	403	212(52.6)	2.11(1.54 , 2.90)	14.77
RRSCombat	No	1587	520(32.8)	Ref	
	Yes	290	175(60.3)	3.21(2.47 , 4.15)	40.94
Indashyikirwa-Rwanda	No	1031	193(18.7)	Ref	
	Yes	629	236(37.5)	2.69(2.14 , 3.37)	36.21
oPt-Palestine	No	277	134(48.4)	Ref	
	Yes	94	67(71.3)	2.83(1.70 , 4.71)	8.09
**Overall**	No	3169	941(29.7)	Ref	
	Yes	1416	690(48.7)	2.53(2.18 , 2.94)	100
**NPSV experience**					
SSCF-SA	No	449	167(37.2)	Ref	
	Yes	228	139(61.0)	2.64(1.90 , 3.67)	26.51
RRS-Combat	No	1823	663(36.4)	Ref	
	Yes	54	32(59.3)	2.82(1.62 , 4.91)	73.49
**Overall**	No	2272	830(36.5)	Ref	
	Yes	282	171(60.6)	2.65(2.02 , 3.46)	100

† All models adjusted for alcohol use and woman's age, except oPt, where model adjusted for age only.

Discussion

VAWG, and poor mental health are complex and overlapping problems, the results from this pooled analysis of data from men and women participating in five IPV prevention studies across Africa and in the Middle East showed evidence of a clear association between poor mental health and men's perpetration of both physical intimate and non-partner sexual violence. Among women, we also found consistent associations between experiencing IPV and NPSV and reports of poor mental health outcomes.

There was a consistent positive association between men's depressive symptomatology and their perpetration of IPV in the pooled analysis, and in four out of the five country level analyses. These findings are similar to those of the UN Multi-country study which showed that men's depression was associated with physical and/or sexual IPV perpetration in three sites across Asia and the Pacific (Bangladesh, Cambodia, and China) (17), and also reflects findings from studies in the Global North(27). For example, in meta-analytic reviews, Stith et al. 2004 and Schumacher et al. 2001 found that depression was a moderate risk factor for male perpetrated violence against female partners(28, 29) In addition, a study in

For peer review only - http://bmjopen.bmj.com/site/about/guidelines.xhtml

the USA found that men with depression showed an increased risk for perpetration of intimate partner violence(30). There are a few potential reasons why men's depressive symptoms may be associated with their perpetration of IPV. It may be that men who are depressed engage in more drinking behaviour, which in turn increases their risk of IPV/NPSV perpetration. This is in keeping with other research that found poor mental health and alcohol are co-morbid and this increased men's risk of violence perpetration (18), as well as research from multiple low to middle income settings which indicate that men's drinking place women at increased risk of IPV and NPSV perpetration (31). It may also be that men who are depressed feel that they are unable to achieve traditional gender-role expectations placed on them, such as economic provision, or having stable employment, and in turn, seek to exercise their gendered power by controlling and dominating their partners. Previous research in informal settlements found that among men in contexts of poverty, unemployment and social marginalisation, controlling their female partners has been used to consolidate hierarchy within social relationships and strengthen their self-evaluation of their performance as men (32).

The reason for the lack of association between depressive symptoms and men's IPV perpetration in oPt is unclear. Previous work has found that exposure to political violence places men at risk for perpetration of IPV(33), and this may mask other associations. Further work is required to understand the interrelationships between exposure to political violence (not measured here), men's mental health, and men's perpetration of IPV.

In this study, the association between men's depressive symptoms and perpetration of NPSV was significant in two of the three studies where NPSV was measured (SSCF and Sonke Change Trials), but not in RRS-COMBAT, the latter finding may be due to small number of men reporting depressive symptoms and non-partner sexual violence perpetration in the RRS-COMBAT intervention. Nonetheless, men who reported depressive symptoms did have an overall increased odds of perpetrating NPSV. An analysis of associations between past year NPSV perpetration and depression of men in a population based sample from Bougainville, Papua New Guinea in contrast did not show associations (34).

We found consistent positive associations between men's reports of PTSD symptoms and perpetration of IPV in 4 of the 5 studies and the pooled analysis, and for men's perpetration of NPSV, where this was significant in the pooled analysis and in all three of the individual studies (SSCF, Sonke and RRS-COMBAT) where NPSV was examined. There remains very little research on the association between PTSD and IPV and NPSV in general population samples. The evidence that there is primarily comes from largely military/offender populations (12, 13), and the analyses of these is complicated by the high rates of

For peer review only - http://bmjopen.bmj.com/site/about/guidelines.xhtml

traumatic experiences that military staff may have experienced and other forms of violence in offender populations. The study of men in Bougainville, Papua New Guinea with very high population prevalence of PTSD and depression only found associations between past year depression and IPV perpetration, not with PTSD ((34). However, findings from our study extend the understanding of PTSD as a risk factor for IPV in general populations by showing that PTSD doubles the risk for NPSV perpetration. This advances the knowledge base, particularly in LMIC country settings, where non-partner sexual violence, particularly in Sub-Saharan African countries are prevalent.

The associations between women's experience of IPV and NPSV and depressive and PTSD symptoms, reflects the broader literature base (10). It also extends the knowledge base to include associations between NPSV experience among women and subsequent experience of PTSD. In addition, PTSD can be complex and often overlaps with depression among women. Therefore, disentangling the dimensions of PTSD and depression remains important to inform prevention programming and health service delivery among women. Prevention interventions need to address the mental health needs of women in prevention and response to IPV and NPSV. (35).

Strengths and Limitations

This study's key strengths is the pooled analysis of data from multiple settings and interventions across low to middle income setting in the global South, using comparable measures of men's IPV and NPSV perpetration, and women's experience of IPV and NPSV, and key covariates such as alcohol use. This addresses the limitation of many previous studies, which tend to focus on IPV only, and use differing measures of drivers and outcomes examining the associations between IPV and NPSV and mental health. The study also adds to the scant evidence base in the global South, where the co-occurring problems of poor mental health and VAWG are prevalent. Furthermore, it highlights the complex and overlapping nature of alcohol, poor mental health as drivers and outcomes of VAWG, often against the backdrop of other structural drivers, such as poverty, food insecurity and unemployment. However, we also recognize limitations in the research. Previous research suggests that there is likely a bi-directional relationship between recent IPV and poor mental health particularly for women (10, 36). However, the cross-sectional nature of the current data limits our ability to draw any conclusions about the temporal relationships, and necessitates future longitudinal analysis. Only two of the five studies were population-based and other studies were limited in generalizability, as they were based on populations recruited for the purpose of impact evaluation. Despite the current limitations, the study confirms that poor mental health are

For peer review only - http://bmjopen.bmj.com/site/about/guidelines.xhtml

important drivers of men's use of violence, and women experiencing IPV and NPSV often experience poor mental health in LIMIC settings.

Conclusion

Our analysis has shown a consistent pattern of depression and PTSD symptoms as drivers of men's perpetration of IPV and NPSV, and increased depression and PTSD symptoms as outcomes among women who are in relationships characterised by IPV and NPSV. Interventions to address VAWG should more actively consider addressing poor mental health as drivers, and address poor mental health outcomes among women experiencing IPV and NPSV in LMIC settings in prevention and treatment programmes.

Acknowledgements

The authors wish to thank the research teams, programme staff, and participants from all studies included in this pooled analysis.

Contributorship statement

LR conceptualized the analysis with AG and RJ. EC led the statistical analysis. AG was a PI on the Stepping Stones and Creating Futures study in South Africa, EC and DOA, and AAL were Co- PIs on the RRS-COMBAT study in Ghana, KD was a PI on the Indashyikirwa study in Rwanda, and MM is a VAWG and mental health researcher, who contributed to the overall drafting of the manuscript. RJ was the Director of the overall What Works to Prevent Violence Against Women and Girls Global programme. LR led the drafting of the manuscript; all authors contributed to comments and revisions.

Funding

All studies presented and this pooled analysis of the data were funded through the What Works to Prevent Violence? A Global Programme on Violence Against Women and Girls (VAWG) funded by the UK Government's Department for International Development (DFID), Grant number: PO 6254. However, the views expressed do not necessarily reflect the department's official policies and the funders had no role in study design; collection, management, analysis, and interpretation of data; writing of the report; and the decision to submit the paper for publication. Funding was managed by the South African Medical Research Council. Time drafting this manuscript was funded by the South African Medical Research Council.

For peer review only - http://bmjopen.bmj.com/site/about/guidelines.xhtml

Competing Interest

There are no potential conflicts of interest to declare.

Data sharing

De- identified individual participant data for Stepping Stones and Creating Futures (South Africa), Sonke Change trial (South Africa), and Evaluation of the RRS-COMBAT intervention (Ghana) and oPt intervention , are available to anyone who wishes to access the data for any purpose at https://medat.samrc.ac.za/index.php/catalog/WW . De- identified individual participant data from the Indashyikirwa couples surveys (Rwanda) are available from the Principal Investigator of the study, Dr Kristin Dunkle: kristin.dunkle@mrc.ac.za , but may require permission from the Rwandan Ministry of Gender and Family Promotion (MIGEPROF) before transfer.

ORCID iD

Leane Ramsoomar: http://orcid.org/0000-0003-1934-579X

Andrew Gibbs: http://orcid.org/0000-0003-2812-5377

Esnat Chirwa: http://orcid.org/ 0000-0003-0471-4978

Kristin Dunkle: http://orcid.org/0000-0003-3071-5544

Rachel Jewkes http://orcid.org/0000-0002-4330-6267

Mercilene Machisa https://orcid.org/0000-0001-7275-1100

Deda Ogum-Alangea http://orcid.org/0000-0001-7496-8514

Adolphina Addo-Lartey https://orcid.org/0000-0001-8852-7189

Ethical issues

Ethical clearance for all studies was obtained prior to the studies commencing. For the Stepping Stones and Creating Futures Trial, clearance was obtained from the South African Medical Research Council's Ethics Committee (EC006-2/2015) and the University of KwaZulu-Natal’s Biomedical Research Ethics Committee (BFC043/15). For the Sonke Change Trial, ethical clearance was obtained from the University of Witwatersrand’s Ethics Committee (M150443). For the Indashyikirwa couple’s intervention in Rwanda, ethical approval was obtained from the Rwandan National Ethics Committee (340/RNEC/2015) and the South Africa Medical Research Council Ethics Committee (EC033-10/2015). A required research permit

For peer review only - http://bmjopen.bmj.com/site/about/guidelines.xhtml

was obtained from the National Institute of Statistics Rwanda (0738/2015/10/NISR). For the RRS-COMBAT intervention in Ghana, the Noguchi Memorial Institute for Medical Research, University of Ghana ((# 006/15–16) and the South African Medical Research Council's Ethics Committee (EC031-9/2015) granted ethical clearance for the study. Ethical clearance for the oPt study was granted by the South African Medical Research Council's ethics committee (EC014-5/2016). All studies followed ethical and safety guidelines for research on violence against women. All participants provided written informed consent before participation. Further information on the studies ethics is available in the study sources referenced in Table 1.

For peer review only

For peer review only - http://bmjopen.bmj.com/site/about/guidelines.xhtml

References

1. World Health Organization. Violence against women prevalence estimates, 2018: global, regional and national prevalence estimates for intimate partner violence against women and global and regional prevalence estimates for non-partner sexual violence against women. 2021.
2. Ellsberg M, Jansen HA, Heise L, Watts CH, Garcia-Moreno C. Intimate partner violence and women's physical and mental health in the WHO multi-country study on women's health and domestic violence: an observational study. The lancet. 2008;371(9619):1165-72.
3. Gibbs A, Dunkle K, Ramsoomar L, Willan S, Jama Shai N, Chatterji S, et al. New learnings on drivers of men's physical and/or sexual violence against their female partners, and women's experiences of this, and the implications for prevention interventions. Global health action. 2020;13(1):1739845.
4. Abramsky T, Watts CH, Garcia-Moreno C, Devries K, Kiss L, Ellsberg M, et al. What factors are associated with recent intimate partner violence? Findings from the WHO multi-country study on women's health and domestic violence. BMC public health. 2011;11(1):109.
5. Ellsberg M, Ovince J, Murphy M, Blackwell A, Reddy D, Stennes J, et al. No safe place: Prevalence and correlates of violence against conflict-affected women and girls in South Sudan. PLoS one. 2020;15(10):e0237965.
6. Stöckl H, Sardinha L, Maheu-Giroux M, Meyer SR, García-Moreno C. Physical, sexual and psychological intimate partner violence and non-partner sexual violence against women and girls: a systematic review protocol for producing global, regional and country estimates. BMJ open. 2021;11(8):e045574.
7. Machisa MT, Christofides N, Jewkes R. Mental ill health in structural pathways to women's experiences of intimate partner violence. PloS one. 2017;12(4):e0175240.
8. Machisa MT, Christofides N, Jewkes R. Structural pathways between child abuse, poor mental health outcomes and male-perpetrated intimate partner violence (IPV). PloS one. 2016;11(3):e0150986.
9. Lagdon S, Armour C, Stringer M. Adult experience of mental health outcomes as a result of intimate partner violence victimisation: a systematic review. European journal of psychotraumatology. 2014;5(1):24794.
10. Bacchus LJ, Ranganathan M, Watts C, Devries K. Recent intimate partner violence against women and health: a systematic review and meta-analysis of cohort studies. BMJ open. 2018;8(7):e019995.
11. Devries KM, Mak JY, Bacchus LJ, Child JC, Falder G, Petzold M, et al. Intimate partner violence and incident depressive symptoms and suicide attempts: a systematic review of longitudinal studies. PLoS medicine. 2013;10(5):e1001439.
12. Bell KM, Orcutt HK. Posttraumatic stress disorder and male-perpetrated intimate partner violence. JAmA. 2009;302(5):562-4.
13. Orcutt HK, King LA, King DW. Male-perpetrated violence among Vietnam veteran couples: relationships with veteran's early life characteristics, trauma history, and PTSD symptomatology. Journal of traumatic stress. 2003;16(4):381-90.
14. Trevillion K, Williamson E, Thandi G, Borschmann R, Oram S, Howard LM. A systematic review of mental disorders and perpetration of domestic violence among military populations. Social psychiatry and psychiatric epidemiology. 2015;50(9):1329-46.
15. Jordan BK, Marmar CR, Fairbank JA, Schlenger WE, Kulka RA, Hough RL, et al. Problems in families of male Vietnam veterans with posttraumatic stress disorder. Journal of consulting and clinical psychology. 1992;60(6):916.

For peer review only - http://bmjopen.bmj.com/site/about/guidelines.xhtml

16. King LA, King DW. Male-perpetrated domestic violence: Testing a series of multifactorial family models. Violence Against Women and Family Violence: Developments in Research, Practice, and Policy. 2004.
17. Fulu E, Jewkes R, Roselli T, Garcia-Moreno C. Prevalence of and factors associated with male perpetration of intimate partner violence: findings from the UN Multi-country Cross-sectional Study on Men and Violence in Asia and the Pacific. The lancet global health. 2013;1(4):e187-e207.
18. Machisa MT. The relationships between poor mental health, child abuse, women's experiences and male perpetration of intimate partner violence in Gauteng province of South Africa. Johannesburg: University of the Witwatersrand; 2019.
19. O'Donnell ML, Creamer M, Pattison P. Posttraumatic stress disorder and depression following trauma: understanding comorbidity. American Journal of Psychiatry. 2004;161(8):1390-6.
20. Hatcher AM, Gibbs A, Jewkes R, McBride R-S, Peacock D, Christofides N. Effect of childhood poverty and trauma on adult depressive symptoms among young men in peri-urban South African settlements. Journal of Adolescent Health. 2019;64(1):79-85.
21. Radloff LS. A self-report depression scale for research in the general population. Applied psychol Measurements. 1977;1:385-401.
22. Blodgett JM, Lachance CC, Stubbs B, Co M, Wu Y-T, Prina M, et al. A systematic review of the latent structure of the Center for Epidemiologic Studies Depression Scale (CES-D) amongst adolescents. BMC psychiatry. 2021;21(1):1-8.
23. Mollica RF, Caspi-Yavin Y, Bollini P, Truong T, Tor S, Lavelle J. The Harvard Trauma Questionnaire: validating a cross-cultural instrument for measuring torture, trauma, and posttraumatic stress disorder in Indochinese refugees. Journal of nervous and mental disease. 1992.
24. García-Moreno C, Jansen HA, Ellsberg M, Heise L, Watts C. WHO multi-country study on women's health and domestic violence against women: World Health Organization; 2005.
25. Jewkes R, Dunkle K, Koss MP, Levin JB, Nduna M, Jama N, et al. Rape perpetration by young, rural South African men: Prevalence, patterns and risk factors. Social science & medicine. 2006;63(11):2949-61.
26. Jewkes R, Fulu E, Tabassam Naved R, Chirwa E, Dunkle K, Haardörfer R, et al. Women's and men's reports of past-year prevalence of intimate partner violence and rape and women's risk factors for intimate partner violence: A multicountry cross-sectional study in Asia and the Pacific. PLoS medicine. 2017;14(9):e1002381.
27. Voith LA, Lee H, Russell K. How Trauma, Depression, and Gender Roles Lead to Intimate Partner Violence Perpetration Among a Sample of Predominately Low-Income Black, Indigenous, Men of Color: A Mixed Methods Study. Journal of Interpersonal Violence. 2022;37(9-10):NP6487-NP513.
28. Stith SM, Smith DB, Penn CE, Ward DB, Tritt D. Intimate partner physical abuse perpetration and victimization risk factors: A meta-analytic review. Aggression and violent behavior. 2004;10(1):65-98.
29. Schumacher JA, Feldbau-Kohn S, Slep AMS, Heyman RE. Risk factors for male-to-female partner physical abuse. Aggression and violent behavior. 2001;6(2-3):281-352.
30. Shorey RC, Febres J, Brasfield H, Stuart GL. The prevalence of mental health problems in men arrested for domestic violence. Journal of family violence. 2012;27(8):741-8.
31. Ramsoomar L, Gibbs A, Chirwa ED, Dunkle K, Jewkes R. Pooled analysis of the association between alcohol use and violence against women: evidence from four violence prevention studies in Africa. BMJ open. 2021;11(7):e049282.
32. Gibbs A, Govender K, Jewkes R. An exploratory analysis of factors associated with depression in a vulnerable group of young people living in informal settlements in South Africa. Global public health. 2018;13(7):788-803.
33. Riggs DS, Caulfield MB, Street AE. Risk for domestic violence: Factors associated with perpetration and victimization. Journal of clinical psychology. 2000;56(10):1289-316.

For peer review only - http://bmjopen.bmj.com/site/about/guidelines.xhtml

34. Jewkes R, Jama-Shai N, Sikweyiya Y. Enduring impact of conflict on mental health and gender-based violence perpetration in Bougainville, Papua New Guinea: a cross-sectional study. Plos one. 2017;12(10):e0186062.
35. Dixon LB, Holoshitz Y, Nossel I. Treatment engagement of individuals experiencing mental illness: review and update. World Psychiatry. 2016;15(1):13-20.
36. Devries KM, Child JC, Bacchus LJ, Mak J, Falder G, Graham K, et al. Intimate partner violence victimization and alcohol consumption in women: A systematic review and meta-analysis. Addiction. 2014;109(3):379-91.

For peer review only

For peer review only - http://bmjopen.bmj.com/site/about/guidelines.xhtml

For peer review only - http://bmjopen.bmj.com/site/about/guidelines.xhtml

BMJ Open

Pooled Analysis of the Association Between Mental Health and Violence Against Women: Evidence from five settings in the global South

Journal:	*BMJ Open*
Manuscript ID	bmjopen-2022-063730.R2
Article Type:	Original research
Date Submitted by the Author:	16-Jan-2023
Complete List of Authors:	Ramsoomar, Leane; South African Medical Research Council, Gender and Health Research Unit; University of Pretoria School of Health Systems and Public Health, School of Health Systems and Public Health<br>Gibbs, Andrew; University of Exeter School of Psychology, Gender and Health Research Unit; South African Medical Research Council, Gender and Health Research Unit<br>Chirwa, Esnat; South African Medical Research Council,<br>Machisa, Mercilene T.; South African Med Res Council, Gender and Health Research Unit<br>Alangea, Deda; University of Ghana School of Public Health, Public Health<br>Addo-Lartey, Adolphina Addoley ; University of Ghana,<br>Dunkle, Kristin ; South African Medical Research Council,<br>Jewkes, Rachel; South African Medical Research Council, Gender and Health Division ; University of the Witwatersrand Johannesburg School of Public Health, School of Public Health
**Primary Subject Heading**:	Public health
Secondary Subject Heading:	Global health
Keywords:	MENTAL HEALTH, Depression & mood disorders < PSYCHIATRY, PUBLIC HEALTH, Substance misuse < PSYCHIATRY, Public health < INFECTIOUS DISEASES, Anxiety disorders < PSYCHIATRY

SCHOLARONE™
Manuscripts

I, the Submitting Author has the right to grant and does grant on behalf of all authors of the Work (as defined in the below author licence), an exclusive licence and/or a non-exclusive licence for contributions from authors who are: i) UK Crown employees; ii) where BMJ has agreed a CC-BY licence shall apply, and/or iii) in accordance with the terms applicable for US Federal Government officers or employees acting as part of their official duties; on a worldwide, perpetual, irrevocable, royalty-free basis to BMJ Publishing Group Ltd ("BMJ") its licensees and where the relevant Journal is co-owned by BMJ to the co-owners of the Journal, to publish the Work in this journal and any other BMJ products and to exploit all rights, as set out in our licence.

The Submitting Author accepts and understands that any supply made under these terms is made by BMJ to the Submitting Author unless you are acting as an employee on behalf of your employer or a postgraduate student of an affiliated institution which is paying any applicable article publishing charge ("APC") for Open Access articles. Where the Submitting Author wishes to make the Work available on an Open Access basis (and intends to pay the relevant APC), the terms of reuse of such Open Access shall be governed by a Creative Commons licence – details of these licences and which Creative Commons licence will apply to this Work are set out in our licence referred to above.

Other than as permitted in any relevant BMJ Author's Self Archiving Policies, I confirm this Work has not been accepted for publication elsewhere, is not being considered for publication elsewhere and does not duplicate material already published. I confirm all authors consent to publication of this Work and authorise the granting of this licence.

For peer review only

Pooled Analysis of the Association Between Mental Health and Violence Against Women: Evidence from five settings in the global South

Leane Ramsoomar[1,2,3,] Andrew Gibbs[1,4], Esnat Chirwa[1,2], Mercilene Machisa[1,2], Deda Ogum Alangea[5], Adolphina Addo-Lartey[5], Kristin Dunkle[1], Rachel Jewkes[1,2]

1 Gender and Health Research Unit, South African Medical Research Council, Pretoria, South Africa

2 School of Public Health, University of the Witwatersrand, Johannesburg, South Africa

3 School of Health Systems and Public Health, University of the Pretoria, Gauteng, South Africa

4 Centre for Rural Health, School of Nursing and Public Health, University of KwaZulu-Natal, Durban, South Africa

5 School of Public Health, University of Ghana

Corresponding author:

Leane Ramsoomar [1,2,3] leane.ramsoomar@mrc.ac.za

[1] Gender and Health Research Unit, South African Medical Research Council, Pretoria, South Africa

[2] School of Public Health, University of the Witwatersrand, Johannesburg, South Africa

[3] School of Health Systems and Public Health, University of the Pretoria, Gauteng, South Africa

For peer review only - http://bmjopen.bmj.com/site/about/guidelines.xhtml

Abstract

Objectives: To describe associations between men's poor mental health (depressive and post-traumatic stress symptomatology) and their perpetration of Intimate Partner Violence (IPV) and non-partner sexual violence (NPSV), and women's mental health and their experiences of IPV and NPSV in five settings in the global South.

Design: A pooled analysis of data from baseline interviews with men and women participating in five Violence against Women and Girls (VAWG) prevention intervention evaluations.

Setting: Three Sub-Saharan African countries [South Africa (2), Ghana, and Rwanda], and one Middle Eastern country, the occupied Palestinian territories (oPt).

Participants: 7021 men and 4,525 women 18+ years old from a mix of self-selecting and randomly selected household surveys.

Main outcome measures: All studies measured depression symptomatology using the Centre for Epidemiological Studies-Depression (CESD), and the Harvard Trauma Scale for PTSD symptoms among men and women. IPV and NPSV were measured using items from modified WHO Women's Health and Domestic Violence and UN Multi-country Study to assess perpetration among men, and experience among women.

Findings: Overall men's poor mental health was associated with increased odds of perpetrating physical IPV and NPSV. Specifically, men who had more depressive symptoms had increased odds of reporting IPV (aOR=2.13 [95%CI 1.58-2.87]) and NPSV perpetration (aOR= (1.62 95% CI 0.97 -2.71) compared to those with fewer symptoms. Men reporting PTSD had higher odds of reporting IPV (aOR= 1.87 [95% CI 1.44 - 2.43]) and NPSV (aOR= 2.13 [95% CI 1.49 -3.05]) perpetration compared to those without PTSD. Women who had experienced IPV (aOR= 2.53 [95% CI 2.18 -2.94]) and NPSV (aOR= 2.65 [95% CI 2.02 -3.46]) had increased odds of experiencing depressive symptoms compared to those who had not.

Conclusions: Interventions aimed at preventing IPV and NPSV perpetration and experience must account for the mental health of men as a risk factor, and women's experience.

For peer review only

Article Summary

Strengths

- Synthesises data across LMIC settings in the global South using comparable measures.
- Addresses the limited geographical scope of studies examining the association between mental health VAWG perpetration and experience in the global South.
- Addresses both IPV and NPSV perpetration by men and experience by women using comparable measures.

Limitations

- Only two of the five studies are population-based, limiting generalizability.
- All data are cross-sectional, limiting inference about causality and direction of effects.

For peer review only - http://bmjopen.bmj.com/site/about/guidelines.xhtml

Introduction

Violence against women and girls (VAWG), particularly, intimate partner violence (IPV) and non-partner sexual violence (NPSV) are significant global public health problems. Globally, about 35% of women report having experienced either physical and/or sexual intimate partner violence or non-partner sexual violence in their lifetime (1). A large body of research has emerged identifying drivers of men's perpetration of physical and or sexual violence against their female partners, and women's experience of this, in diverse contexts and populations. Drivers of violence perpetration and experience include poverty, gender inequalities, normalization of violence, substance use, exposure to abuse as a child, and poor communication in relationships and conflict skills, as well as living conflict-affected settings (2-5). Research has established associations between experience of IPV and a range of negative health outcomes, particularly mental health outcomes, HIV, low birthweight, termination of pregnancy, suicidal ideation and harmful alcohol use (1, 6).

Poor mental health is increasingly recognized as both an outcome and a driver of VAWG (7, 8). Among women, the associations between VAWG and negative health outcomes have been recognised for some time and poor mental health has largely been established as an outcome of IPV and NPSV experience (9). The WHO multi-country study on Women's Health and Domestic violence found significant associations between lifetime experiences of partner violence and self-reported poor health, including more emotional distress, suicidal thoughts and attempts among abused, compared with non-abused, women (2). A systematic review and meta-analysis of cohort studies found a strong association between recent IPV experience and depressive symptoms among women(10). Research has shown that poor mental health may also be a risk factor for women's IPV experience. For instance, Devries and colleagues found that women who experience depression may be more accepting of partners with traits that predispose them to violence (11), while other research found that women who experience depression may withdraw or display lethargy, which impacts their ability to seek help, or remove themselves from relationships (10). Furthermore, the systematic review of cohort studies (mentioned above), found evidence of a bi-directional relationship between depression and IPV experience, whereby women's depressive symptoms were associated with subsequent IPV experience(10).

Among men, research has found that poor mental health is associated with IPV perpetration, but the majority of this evidence has come largely from military or male offender samples from high income countries (12-14). There is, however, heterogeneity among samples and measures and definitions of IPV in previous work. Additionally this prior research has often focused on symptoms of PTSD (8, 13, 15, 16),

For peer review only - http://bmjopen.bmj.com/site/about/guidelines.xhtml

For peer review only

with evidence indicating strong associations between PTSD and IPV perpetration (13). Depression as a risk factor for male perpetration of violence against women has been less frequently studied and where examined, results have been mixed. For example, the cross-sectional population-based UN multi-country study in the Asia-Pacific region found that depression was associated with men's physical and sexual IPV perpetration in samples from four of six countries (17). However, evidence from a systematic review of population based studies found no association between depression and IPV perpetration (18). This may be due to limited numbers of population-based studies on men's violence perpetration, and the frequent omission of measures of men's mental health in studies of IPV perpetration. To our knowledge the association between men's poor mental health and their risk of NPSV perpetration has not been examined in LMICs. However, IPV and NPSV are distinct types of violence, and where data are available, it is important to examine them both to investigate whether there are shared risk factors for men's perpetration of these and, to understand the physical and mental health outcomes for women experiencing them.

Poor mental health often occurs against the backdrop of poverty, adverse childhood experiences, life traumatic events, low education and gender inequality and conflict and the wider context of generalized violence (3, 8, 19, 20). These factors operate either directly or indirectly to impact on childhood adverse events, poor mental health, substance use and relationship conflict, which in turn increase risk for IPV (3). Data from a population-based survey in South Africa found that experiencing adverse childhood events (physical, sexual and emotional abuse) increased the risk of men's poor mental health (PTSD, depression), harmful alcohol use, which in turn increased the likelihood of male-perpetrated IPV (8).

Although poor mental health has been recognized as both a driver and outcome of VAWG, the majority of studies are from high-income settings, focus largely on IPV only, and use differing measures of exposure and outcomes, which limits comparison across settings. Having comparable measures is foundational for making meaningful national, regional and global comparisons and understanding where prevalence and outcomes may differ (21). In low-middle income settings, where co-occurring public health challenges of VAWG, poor mental health and harmful alcohol use are prevalent, there is much less research on the associations between VAWG, poor mental health and VAWG. To strengthen the evidence base from low- and middle-income countries, and advance the current literature base on the role of poor mental health in both IPV and NPSV perpetration and victimisation, we undertook a pooled analysis of baseline data using comparable measurement methods from five IPV prevention studies conducted in

For peer review only

three countries in Africa (South Africa – which had two studies - Ghana and Rwanda) and one conflict-afflicted country in the Middle East, the occupied Palestinian Territories (oPt) [West Bank and Gaza]. All studies report on recent (past 12 month) IPV and NPSV experience/perpetration, as opposed to lifetime exposure to IPV and NPSV. The paper aims to answer the following research questions (i) Is men's poor mental health associated with IPV and NPSV perpetration ? (ii) Is women's experience of IPV and NPSV associated with poor mental health outcomes?.

Methods

The studies included in this pooled analysis were conducted under the UK-Aid funded What Works to Prevent Violence Against Women and Girls? Global Programme (What Works). The primary goal of What Works was to advance the evidence base on the prevalence and drivers of VAWG in the Global South, and determine the effectiveness of interventions to prevent VAWG. The current study used the baseline data from 7021 men and 4,525 women from five VAWG prevention studies in four countries (South Africa, Ghana, Rwanda, and occupied Palestinian Territories [West Bank and Gaza]) to assess the association between poor mental health (depression and PTSD symptomatology) and IPV and NPSV perpetration among men and the association between poor mental health and IPV and NPSV experience among women. These studies include the Stepping Stones and Creating Futures intervention (South Africa), the Sonke Change trial (South Africa), the Rural Response System (RRS-COMBAT) community intervention (Ghana), the Indashyikirwa couples intervention (Rwanda), and a population-based nationally representative survey in the occupied Palestinian Territory (oPt) [West Bank and Gaza]. Further information on the studies is available in the study sources referenced in Table 1.

Patient and public involvement

Patients and the public were not involved in the study design, implementation, or analysis presented in this paper. However, the individual projects had different levels of participant and public involvement as part of the wider What Works research uptake strategy.

For peer review only - http://bmjopen.bmj.com/site/about/guidelines.xhtml

1 Table 1: **Datasets used for men and women included in the pooled analysis**

#	Study	Country	Study Design	# clusters	N (Men)	N (Women)	Sampling or recruitment strategy	Age
1	Evaluation of Stepping Stones and Creating Futures	South Africa	CRCT	34	674	677	Study volunteers	18-35
2	Evaluation of Sonke Change trial	South Africa	CRCT	18	2406	-	Household-based random sample survey	18-45
3	Evaluation of the RRS-COMBAT community intervention	Ghana	CRCT	40	1973	1877	Household-based random sample survey	18+ (men) 18-45 (women)
4	Indashyikirwa -- couples intervention	Rwanda	CRCT	28	1651	1660	Volunteer recruitment from savings and loan association groups	18-50
5	Evaluation of Innovative Media to End VAWG through Community Education and Outreach in the oPt	Occupied Palestinian Territories	Population-based survey	55	308	371	Population-based nationally representative survey	18+ (men) 18+ (women)

For peer review only

Measures

All study measures are presented in Table 2. We assessed socio-demographics for men and women, including, age, current marital status, relationship residence status, and education, employment in the past 3 months and past year, respectively. All studies except Indashyikirwa (couples) asked whether participants had worked in the past 3-months or the past year respectively; the two South African studies asked whether female participants had worked in the past 3-months or not. The purpose of the past 3 month employment question was to give us an indication of recent employment. Income assessments in Indashyikirwa couples were not included in the present analyses, as they were tailored to the local context in which most participants engaged in subsistence agriculture.

Depression symptoms were measured using the previously validated Center for the Epidemiological Studies of Depression Short Form (CESD-10) (22, 23). PTSD symptoms were measured in three of the five studies (SSCF, Sonke Change trial, Ghana (men only), using the previously validated Harvard Trauma questionnaire (24), in settings where it was anticipated that the intervention would impact it. The Harvard Trauma Scale is a widely used cross cultural measure to measure symptoms of Post-traumatic Stress (24, 25), and which has been used to measure PTSD symptoms in low to middle income settings (8, 26).

We measured men's current alcohol use using one item which asked men, "Have you drunk alcohol in the past 12 months?. Responses were either "Yes" or "No"." This is in keeping with international guidelines, which consistently measure current alcohol use as drinking at least one alcoholic drink in the 12 months preceding the baseline data collection(27-29), while women (as a proxy) were asked if they had seen their partner drunk, and how frequently they saw them drunk in the past 12 months, as we did not always have access to the partner, and this was the most reliable measure of partner drunkenness in the past 12 month recall period. Violence perpetration (IPV and NPSV) and experience (IPV) were measured using items from the WHO Women's Health and Domestic Violence survey (30), modified appropriately to assess men's perpetration (17). The NPSV scale was first developed in South Africa (31) and subsequently refined and used extensively in the Asia-Pacific region (32). There was no question on NPSV perpetration in the Indashyikirwa couples or the oPt studies, because of concerns about the particular sensitivity of the questions in those contexts.

For peer review only - http://bmjopen.bmj.com/site/about/guidelines.xhtml

Table 2: **Key Measures**

CONSTRUCT	Indicator	Definition
**VIOLENCE AGAINST WOMEN**	Physical IPV perpetration	Five items used to measure men's physical IPV perpetration in the last 12 months e.g how many times (1) did you slap your current or previous girlfriend or wife or throw something at her which could hurt her? (2) have you pushed or shoved a current or previous girlfriend or wife? (3) have you hit a current or previous girlfriend or wife with a fist or with something else which could hurt her? (4) did you kick, drag, beat, choke or burn a previous or current girlfriend, partner or wife? (5) did you threaten to use or actually use a gun, knife or other weapon against a previous or current girlfriend, partner or wife? Responses: "Never", "Once" "A few times" or "Many times". Men who responded "Once" or more to one or more items were coded as perpetrating physical IPV. The items were developed during the WHO Women's Health and Domestic Violence survey, and UN Multi-country study on Men and Violence in Asia and the Pacific, modified to assess men's perpetration of physical and NPSV in the past year(17, 30).
	Physical IPV experience	Five items to measure women's violence experience in the past 12 months e.g. (1) how many times has a current or previous husband (or boyfriend) slapped you or thrown something at you which could hurt you? (2) pushed or shoved you? (3) hit you with a fist or with something else which could hurt you? (4) kicked, dragged, beaten, choked or burnt you? (5) threatened to use, or actually used, a gun, knife or other weapon against you?(30) Responses: "Never", "Once" "A few times" or "Many times". Women who responded "Once" or more to one or more items were coded as experiencing physical IPV.
	NPSV experience	Six items about NPSV perpetration in the past 12 months e.g. (1) How many times has any man who is NOT your boyfriend or husband forced or persuaded you to have sex against your will?... (2)tried to force you to have sex against your will and did not succeed?... (3) forced you to have sex against your will when you were too drunk or drugged to refuse? (4) did two or more men force you to have sex with them at the same time against your will? (5) did two or more men force you to have sex with them at the same time against your will when you were too drunk or drugged to refuse? (6) was there an occasion when you agreed to have sex with one man and one or more others who you had not agreed to have sex with forced you to have sex with them as well? This was coded in the same way as for the men.
**ALCOHOL USE**	Current Alcohol Use (past 12 months)	Current alcohol use by men was measured by asking one question about alcohol use in the 12 months preceding the baseline data collection: (1) Have you drunk alcohol in the past 12 months?. Responses were either "Yes" or "No".
	Seen partner drunk	1 item assessing if women has seen their partner drunk in the past 12 months. Responses: "Yes" or "No"
	Frequency of seeing partner drunk	1 item assessing how often women had seen their partner drunk in the past 12 months. Responses were "every day or nearly every day" "Weekly" " Once a month" " Less than once a month" " Never" and recoded into: "never" "occasionally" "Frequently"

For peer review only - http://bmjopen.bmj.com/site/about/guidelines.xhtml

**MENTAL HEALTH**	Depression symptoms (men and women)	Three studies (SSCF, Sonke Change Trial and RRS-COMBAT) used the 20-item Centre for Epidemiological Studies Depression (CESD) scale used to measure depression symptoms. Items were framed around statements about feelings in the past week, such as: 'During the past week I felt fearful' with responses, 'none or rarely', 'some or a little', 'moderate amount of time', and 'most or all the time'. Items were summed, and ranged between 0-41, with higher scores indicating more depressive symptoms (Cronbach alpha ranged from 0.78-0.91 across studies). Three studies (Indashyikirwa and oPt) used the 10-item Centre for Epidemiological Studies Depression (CESD) short form scale to measure depression symptoms. Items were summed, and ranged between 0-21, with higher scores indicating more depressive symptoms.
	Post-Traumatic Stress Disorder (PTSD) symptoms (men and women).	Harvard Trauma Scale for PTSD, which reflected reactions to traumatic experiences in their lifetime experienced in the past week, such as: "In the past week have you had recurrent thoughts or memories of the most hurtful or terrifying events" or ". In the past week have you had recurrent nightmares". Responses were rated 0= not at all, to 3= extremely. Items were summed with higher scores indicating more PTSD symptoms. Cronbach alphas ranged from 0.91-0.94 across studies where PTSD was measured.

For peer review only - http://bmjopen.bmj.com/site/about/guidelines.xhtml

Data analysis

Descriptive statistics (frequencies and percentages) were used to summarize participants' socio-demographic characteristics within each study and in the pooled analysis. Within-study and pooled estimates considered any clustering with each study's sampling procedures. All pooled estimates were weighted according to the study sample size. We used forest plots, I-square and Cochran's Q statistics to assess the consistency of outcomes across the studies. The I-square values showed high heterogeneity in physical IPV as an outcome (80%, p-value<0.001 for men) and low heterogeneity for depression symptoms among women (14.8%, p-value=0.329). We used mixed-effects models to estimate overall effects and account for any heterogeneity across the studies due to methodological diversity. We fitted a one-stage Individual Patient Data (IPD) meta-analysis using mixed-effects logistic regression models to account for within-and between-study variances (heterogeneity) across studies for both men and women (17). We derived study-specific estimates and forest plots from a post-estimation model of the mixed-effects logistic regression model. Both the main and post-estimation models included participants' age as fixed effects. Prior to fitting models and in order to reduce any bias due to missing data, we examined patterns and levels of missing data in all key variables such as physical IPV, depression score, PTSD and non-partner sexual violence. No missing data was encountered in the key variables for the women's datasets. In the men's datasets, we found no systematic patterns in the missing data and amount of missing data was minimal and ranged from 0.16% (depression score) to 1.3% (PTSD score). We then used full information maximum likelihood to handle missing data in the mixed effect models. We conducted sensitivity analysis to assess the impact of including the experience of childhood trauma on model estimates for studies that measured childhood trauma. We found non-significant change in model estimates. Thus, the final models were adjusted for participants' age and alcohol use or partner alcohol use (for women), because of the association between age and IPV experience/perpetration, and co-morbidity between alcohol, VAWG and poor mental health found in previous research. All data were analyzed using Stata Statistical Software: Release 17. College Station, TX: StataCorp LLC. StataCorp. 2019.

Results

Men

The mean age of men across all studies was 33.1 years old (SD=11.4). Forty-five percent of men were married while 45% were in a non-marital relationship. Overall, only 10% of men across all studies were not in a relationship; 61% of men indicated they were living with their partners, except in Rwanda (100%) couples and oPt (95%) where the majority were married. Almost a third of men (29%) reported not living

For peer review only - http://bmjopen.bmj.com/site/about/guidelines.xhtml

together with their female partners. Most of the men had secondary school education or above (66%) and half (53%) the men in three studies were employed in the past 3 months (we did not ask about employment status in Rwanda and the oPt). (Table 3)

Women

The mean age of women across the four studies was 31.3 years old (SD=8.4). Over half (56%) of women were married, 38% were in a relationship, 75% were living with their partner or spouse and 19% were not living with their partner, and 6% were not in a relationship. Nearly one-half (48%) of the women had completed secondary school education or above, and 53% reported working in the past 3 months, though this was not asked in Rwanda or oPt. Just over a third of women (32%) reported that their partner consumed alcohol in the past month. (See Table 3)

For peer review only

For peer review only - http://bmjopen.bmj.com/site/about/guidelines.xhtml

Table 3: **Socio-demographics, prevalence of alcohol consumption among men, reports of partner alcohol consumption among women in the included studies**

	**Stepping Stones and Creating Futures Trial, South Africa**	**Sonke Change Trial, South Africa**	**RRS COMBAT, Ghana**	**Indashyikirwa - couples, Rwanda**	**oPt (Palestine)**	**All studies**
	**n(%) /mean(sd)**	**n(%)/mean(sd)**	**n(%) /mean(sd)**	**n(%) /mean(sd)**	**n(%) /mean(sd)**	**n(%) /mean(sd)**
**MEN**	**(n=674)**	**(n=2406)**	**(n=1973)**	**(n=1651)**	**(n=317)**	**(n=7021)**
**Age**	23.8(3.6)	27.6(5.7)	39.3(14.9)	35.6(7.1)	43.9(13.8)	33.1(11.4)
**Current marital status**						
Married	22(3.3)	448(18.6)	1271(64.4)	1095(66.3)	301(95.0)	3137(44.7)
In a relationship	508(75.4)	1539(64.0)	533(27.0)	556(33.7)	0(0)	3136(44.7)
No relationship	144(21.4)	391(16.2)	169(8.6)		16(5.0)	720(10.3)
missing	0(0)	28(1.2)	0(0)			28(0.4)
**Relationship residence status**						
Living together	73(10.8)	932(38.7)	1314(66.6)	1651(100)	301(95.0)	4271(60.8)
Not living together	457(67.8)	1055(43.9)	490(24.8)		0(0)	2002(28.5)
No relationship	144(21.4)	391(16.2)	169(8.6)		16(5.0)	720(10.3)
missing	0(0)	28(1.2)	0(0)			28(0.4)
**Education**						
none	0(0)	0(0)	372(18.9)	265(16.1)	14(4.4)	651(9.3)
Primary school	77(11.4)	140(5.8)	334(16.9)	1088(65.9)	75(23.7)	1714(24.4)
Sec school or above	597(88.6)	2249(93.5)	1267(64.2)	298(18)	227(71.6)	4638(66.1)
missing	0(0)	17(0.7)	0(0)		1(0.3	18(0.3)
**Employed in past 3 months**	240(35.7)	1192(50)	1231(71.6)	n/m*	n/m	2663(52.7)
**Employed in past year**	156(23.2)	806(33.7)	1251(63.4)	n/m	n/m	2213(43.8)
**Drinks alcohol**	294(43.6)	948(39.4)	291(15.0)	246(15.0)	n/m	1779(27.0)

For peer review only - http://bmjopen.bmj.com/site/about/guidelines.xhtml

WOMEN	(n=677)	-	(n=1877)	(n=1660)	(n=371)	(n=4585)
**Age in yrs. – mean(sd)**	23.9(3.6)		31.4(8.5)	32.7(6.6)	37.7(11.7)	31.3(8.4)
**Current marital status**						
Married	29(4.28)	-	1068(56.9)	1096(66)	365(98.4)	2558(55.8)
In a relationship	524(77.4)	-	664(35.4)	564(34)	0(0)	1752(38.2)
No relationship	124(18.3)	-	145(7.7)	0(0)	6(1.6)	275(6)
**Relationship residence status**						
Living together	113(16.7)	-	1303(69.4)	1660(100)	365(98.4)	3441(75.1)
Not living together	440(65.0)	-	429(22.9)	0(0)	0(0)	869(19)
No relationship	124(18.3)	-	145(7.7)	0(0)	6(1.6)	275(6)
**Education**						
None	0(0)	-	401(21.4)	288(17.4)	3(0.8)	692(15.1)
Primary school	56(8.3)	-	426(22.7)	1115(67.2)	120(32.4)	1717(37.5)
Sec school or above	621(91.7)	-	1050(55.9)	257(15.5)	248(66.8)	2176(47.5)
**Employed in past 3 months**	173(25.6)	-	1174(62.7)	n/m	n/m	1347(52.8)
**Employed in past year**	97(14.3)	-	1034(55.1)	n/m	n/m	1131(44.3)
**Seen Partner frequently drunk in past year**	357(52.7)		268(14.3)	742(44.7)	n/m	1367(32.4)
Never	320 (47.3)	-	1609 (85.7)	918 (55.3)	n/m	2847
Occasionally	220 (32.5)	-	115 (6.1)	471 (28.4)	n/m	806
Frequently	137 (20.2)	-	153 (8.2	271 (16.3)	n/m	561

* not measured

\- Men only sample

For peer review only

For peer review only - http://bmjopen.bmj.com/site/about/guidelines.xhtml

IPV and NPSV perpetration, poor mental health and alcohol consumption among men

Among the 7,021 men across the studies, almost a third (29%) reported perpetrating past year intimate partner violence, with study-specific prevalence ranging from 12% (Ghana) to 50% (SSCF). Across the three studies where men were asked about perpetration of NPSV, 26% of men reported perpetrating past year NPSV, ranging from 10% in Ghana to 39% in SSCF. We did not ask about the perpetration of NPSV in Rwanda or oPt studies, as we were advised by the local partner that these questions would be too sensitive for these socio-political contexts. Overall, 29% of men reported depressive symptoms (range: 18% to 46%), and in the three studies where we asked about PTSD symptomatology (SSCF, Sonke Change trial and Ghana), 6% reported PTSD (range: 5-14.2%). In four studies where alcohol was asked about, 68% of men reported alcohol consumption in the past year (SSCF); 64% in the Sonke Change Trial; 44% in RRS-COMBAT, and 37% in Indashyikirwa. Alcohol consumption was not included in the questionnaire in the oPt. (See Table 4)

Women

IPV and NPSV experience, poor mental health, and reports of partner alcohol consumption

Among the 4,585 women across the studies, just over a third (31%) reported experiencing past year intimate partner violence, with study-specific prevalence ranging from 16% (Ghana) to 60% (SSCF). Across the two studies where NPSV was reported (SSCF and RRS-COMBAT), 11% of women reported experiencing past year NPSV, ranging from 3% in Ghana to 34% in SSCF. Overall, 36% of women reported depressive symptoms (range: 26% to 45%). Only women in SSCF were asked about PTSD symptomatology, and 21% reported such symptoms. (See Table 4)

For peer review only - http://bmjopen.bmj.com/site/about/guidelines.xhtml

Table 4: **Prevalence of Depression, PTSD symptomatology, Physical IPV and NPSV perpetration among men and experience among women in the studies included**

		Depression	PTSD	Physical IPV	NPSV
**MEN**					
	**N**	**n(%)**	**n(%)**	**n(%)**	**n(%)**
SSCF-SA	674	313(46.4)	96(14.2)	337(50.0)	261(38.7)
Sonke-SA	2406	700(29.1)	126(5.0)	952(39.6)	834(34.7)
RRS COMBAT-Ghana	1973	619(31.4)	71(3.60)	235(11.9)	196(9.9)
Indashyikirwa-Rwanda	1651	290(17.6)	n/m	402(24.4)	n/m*
oPt-Palestine	308	112(35.3)	n/m	73(23.0)	n/m
**Overall**	**7021**	**2034(29.0)**	**293(5.8)**	**1999(28.5)**	**1291(25.5)**
**WOMEN**					
	**N**	**Depression**	**PTSD**	**Physical IPV**	**NPSV**
SSCF-SA	677	306(45.2)	142(21.0)	403(59.5)	228(33.7)
RRS COMBAT-Ghana	1877	695(37.0)	*n/m	290(15.5)	54(2.88)
Indashyikirwa-Rwanda	1660	429(25.8)	n/m	629(37.9)	n/m
oPt-Palestine	371	201(54.2)	n/m	94(25.3)	n/m
**Overall**	**4585**	**1631(35.6)**	**142(21.0)**	**1416(30.9)**	**282(11.0)**

* not measured

For peer review only - http://bmjopen.bmj.com/site/about/guidelines.xhtml

Men

Association between poor mental health and men's violence perpetration

The association between men's depressive symptomatology and perpetration of past year IPV was significant across four studies, except for oPt, where the association was only suggestive (p=0.14).(Table 5). The odds ranged from aOR=1.56 [95% CI 1.17-2.07] in the RRS-COMBAT sample to aOR=3.45 [95% CI 2.64- 4.52] in the Indashyikirwa sample. In the pooled analysis of all five studies, having depressive symptoms increased the odds for physical IPV perpetration among men aOR=2.13 [95% CI 1.58-2.87]. In the three studies in which we asked about NPSV perpetration, the association between men's depressive symptomatology and NPSV perpetration was significant across the two South African studies (SSCF aOR=1.93 [95%CI 1.41- 2.64] and the Sonke Change Trial aOR=3.01[95%CI 2.51-3.62), but not the RRS-COMBAT study in Ghana. In the pooled analysis (3 studies), having depressive symptoms increased the odds for non-partner sexual violence perpetration more than one half (aOR=1.62 [95%CI 0.97- 2.71]), but this was not significant (p=0.06).

In the three studies where PTSD was measured, there were significant associations between men's PTSD symptomatology and past year physical IPV perpetration. Across individual studies (SSCF, Sonke Change Trial and RRS-COMBAT) the adjusted association for PTSD symptomatology and past year IPV perpetration ranged from aOR=1.79 [95%CI 1.15-2.79] in SSCF to aOR=2.51[95%CI 1.74-3.62] in the Sonke Change Trial (Table 5). In the pooled analysis among men who reported PTSD, the odds of perpetrating past year physical IPV were increased almost two-fold (aOR= 1.87 [95%CI 1.44-2.43]), compared to those who did not report PTSD symptoms. Post-traumatic Stress Disorder (PTSD) symptomatology was also significantly associated with NPSV perpetration across all three studies, with adjusted associations ranging from aOR=1.70 [95% CI 1.10-2.62] in the Stepping Stones and Creating Futures Trial to aOR=3.08 [95%CI 2.13-4.45] in the Sonke Change Trial. In the pooled analysis, the odds of perpetrating past year non-partner sexual violence were increased more than two-fold among men who reported PTSD aOR= 2.13 [95%CI 1.49-3.05] compared to those who did not report PTSD symptoms.

For peer review only - http://bmjopen.bmj.com/site/about/guidelines.xhtml

Table 5: Association between depression and PTSD symptoms and perpetration Physical IPV and Non-partner sexual violence

		All (N)	Past year physical IPV perpetration			Past year NPSV perpetration		
			n(%)	aOR(95% CI)†	Study weight	n(%)	aOR(95% CI)	Study weight
	**Depression**							
SSCF-SA	No	356	149(41.8)	Ref		113(31.7)	Ref	
	Yes	313	188(60.1)	2.14(1.57 , 2.91)	9.58	148(47.3)	1.93(1.41 , 2.64)	13.31
Sonke-SA	No	1695	534(31.5)	Ref		462(27.3)	Ref	
	Yes	698	416(59.6)	3.22(2.68 , 3.87)	34.17	371(53.0)	3.01(2.51 , 3.62)	47.42
RRS COMBAT-Ghana	No	1354	145(10.7)	Ref		139(10.3)	Ref	
	Yes	619	90(14.5)	1.56(1.17 , 2.07)	28.27	57(9.2)	0.89(0.64 , 1.23)	39.26
Indashyikirwa-Rwanda	No	1357	271(20.0)	Ref		n/m	n/m	n/m
	Yes	289	131(45.3)	3.45(2.64 , 4.52)	23.57	n/m	n/m	n/m
oPt-Palestine	No	198	41(20.7)	Ref		n/m	n/m	n/m
	Yes	110	32(29.1)	1.46(0.84 , 2.52)	4.41	n/m	n/m	n/m
**Overall**	No	4960	1140(23.0)	Ref		714(21.0)	Ref	
	Yes	2029	857(42.2)	2.13(1.58 , 2.87)	100	576(35.3)	1.62(0.97 , 2.71)	100
	**PTSD**							
SSCF-SA	No	572	276(48.2)	Ref		212(37.1)	Ref	
	yes	96	60(62.5)	1.79(1.15 , 2.79)	13.46	48(50.0)	1.70(1.10 , 2.62)	13.46
Sonke-SA	No	2204	833(37.8)	Ref		727(33.0)	Ref	
	Yes	126	76(60.3)	2.51(1.74 , 3.62)	46.8	76(60.3)	3.08(2.13 , 4.45)	46.8
RRS COMBAT- Ghana	No	1902	220(11.6)	Ref		180(9.5)	Ref	
	Yes	71	15(21.1)	2.05(1.14 , 3.68)	39.75	16(22.5)	2.78(1.56 , 4.96)	39.75
Overall	No	4678	1329(28.4)	Ref		1119(23.9)	Ref	
	Yes	293	151(51.5)	1.87(1.44 , 2.43)	100	140(47.8)	2.13(1.49 , 3.05)	100

† All models adjusted for participants age only

For peer review only - http://bmjopen.bmj.com/site/about/guidelines.xhtml

Among women across all studies, experiencing IPV in the past 12 months was significantly associated with reported depressive symptomatology with adjusted associations ranging from aOR= 2.11 [95% CI 1.54-2.90] in SSCF to aOR= 3.21 [95% CI 2.47-4.15] in RRS-COMBAT. In the pooled analysis, women who experienced IPV in the past 12 months, compared to those who had not, had a two and a half fold increased odds of experiencing depressive symptoms than those who did not aOR= 2.53 [95% CI 2.18-2.94].

Similarly, among women who had experienced NPSV in the past 12 months there were significant associations with depressive symptoms, with the adjusted associations ranging from aOR=2.64 [95% CI 1.90-3.67] in the SSCF to aOR= 2.82 [95% CI 1.62-4.91] in the RRS-COMBAT to. In the pooled analysis, women who experienced NPSV in the past 12 months had a more than two and a half fold increased odds of experiencing depressive symptoms than those who did not aOR= 2.65 [95% CI 2.02-3-46]. (See Table 6).

For peer review only - http://bmjopen.bmj.com/site/about/guidelines.xhtml

Table 6: **Association between IPV and NPSV and experience and depressive symptoms among women**

			Depression		
		All(N)	n(%)	aOR(95%CI) †	Study weight
**Physical IPV experience**					
SSCF-SA	No	274	94(34.3)	Ref	
	yes	403	212(52.6)	2.11(1.54 , 2.90)	14.77
RRSCombat	No	1587	520(32.8)	Ref	
	Yes	290	175(60.3)	3.21(2.47 , 4.15)	40.94
Indashyikirwa-Rwanda	No	1031	193(18.7)	Ref	
	Yes	629	236(37.5)	2.69(2.14 , 3.37)	36.21
oPt-Palestine	No	277	134(48.4)	Ref	
	Yes	94	67(71.3)	2.83(1.70 , 4.71)	8.09
**Overall**	No	3169	941(29.7)	Ref	
	Yes	1416	690(48.7)	2.53(2.18 , 2.94)	100
**NPSV experience**					
SSCF-SA	No	449	167(37.2)	Ref	
	Yes	228	139(61.0)	2.64(1.90 , 3.67)	26.51
RRS-Combat	No	1823	663(36.4)	Ref	
	Yes	54	32(59.3)	2.82(1.62 , 4.91)	73.49
**Overall**	No	2272	830(36.5)	Ref	
	Yes	282	171(60.6)	2.65(2.02 , 3.46)	100

† All models adjusted for alcohol use and woman's age, except oPt, where model adjusted for age only.

Discussion

VAWG, and poor mental health are complex and overlapping problems, the results from this pooled analysis of data from men and women participating in five IPV prevention studies across Africa and in the Middle East showed evidence of a clear association between poor mental health and men's perpetration of both physical intimate and non-partner sexual violence. Among women, we also found consistent associations between experiencing IPV and NPSV and reports of poor mental health outcomes.

There was a consistent positive association between men's depressive symptomatology and their perpetration of IPV in the pooled analysis, and in four out of the five country level analyses. These findings are similar to those of the UN Multi-country study which showed that men's depression was associated with physical and/or sexual IPV perpetration in three sites across Asia and the Pacific (Bangladesh, Cambodia, and China) (17), and also reflects findings from studies in the Global North(33). For example, in two meta-analyses, Stith et al. 2004 and Schumacher et al. 2001 found that depression was a moderate risk factor for male perpetrated violence against female partners(34, 35). In addition, a study in the USA

For peer review only - http://bmjopen.bmj.com/site/about/guidelines.xhtml

found that men with depression showed an increased risk for perpetration of intimate partner violence(36).There are a few potential reasons why men's depressive symptoms may be associated with their perpetration of IPV. It may be that men who are depressed engage in more drinking behaviour, which in turn increases their risk of IPV/NPSV perpetration. This is in keeping with other research that found poor mental health and alcohol are co-morbid and this increased men's risk of violence perpetration (18), as well as research from multiple low to middle income settings which indicate that men's drinking place women at increased risk of IPV and NPSV perpetration(37). It may also be that men who are depressed feel that they are unable to achieve traditional gender-role expectations placed on them, such as economic provision, or having stable employment, and in turn, seek to exercise their gendered power by controlling and dominating their partners. Previous research in informal settlements found that among men in contexts of poverty, unemployment and social marginalisation, controlling their female partners has been used to consolidate hierarchy within social relationships and strengthen their self-evaluation of their performance as men (38).

The reason for the lack of association between depressive symptoms and men's IPV perpetration in oPt is unclear. Previous work has found that exposure to political violence places men at risk for perpetration of IPV(39), and this may mask other associations. Further work is required to understand the interrelationships between exposure to political violence (not measured here), men's mental health, and men's perpetration of IPV.

In this study, the association between men's depressive symptoms and perpetration of NPSV was significant in two of the three studies where NPSV was measured (SSCF and Sonke Change Trials), but not in RRS-COMBAT, the latter finding may be due to small number of men reporting depressive symptoms and non-partner sexual violence perpetration in the RRS-COMBAT intervention. Nonetheless, men who reported depressive symptoms did have an overall increased odds of perpetrating NPSV. An analysis of associations between past year NPSV perpetration and depression of men in a population based sample from Bougainville, Papua New Guinea in contrast did not show associations (40).

We found consistent positive associations between men's reports of PTSD symptoms and perpetration of IPV in 4 of the 5 studies and the pooled analysis, and for men's perpetration of NPSV, where this was significant in the pooled analysis and in all three of the individual studies (SSCF, Sonke and RRS-COMBAT) where NPSV was examined. There remains very little research on the association between PTSD and IPV and NPSV in general population samples. The evidence that there is primarily comes from largely military/offender populations (12, 13), and the analyses of these is complicated by the high rates of

For peer review only - http://bmjopen.bmj.com/site/about/guidelines.xhtml

traumatic experiences that military staff may have experienced and other forms of violence in offender populations. The study of men in Bougainville, Papua New Guinea with very high population prevalence of PTSD and depression only found associations between past year depression and IPV perpetration, not with PTSD ((40). However, findings from our study extend the understanding of PTSD as a risk factor for IPV in general populations by showing that PTSD doubles the risk for NPSV perpetration. This advances the knowledge base, particularly in LMIC country settings, where non-partner sexual violence, particularly in Sub-Saharan African countries are prevalent.

The associations between women's experience of IPV and NPSV and depressive and PTSD symptoms, reflects the broader literature base (10). It also extends the knowledge base to include associations between NPSV experience among women and subsequent experience of PTSD. In addition, PTSD can be complex and often overlaps with depression among women. Therefore, disentangling the dimensions of PTSD and depression remains important to inform prevention programming and health service delivery among women. Prevention interventions need to address the mental health needs of women in prevention and response to IPV and NPSV. (41).

Strengths and Limitations

This study's key strengths is the pooled analysis of data from multiple settings and interventions across low to middle income setting in the global South, using comparable measures of men's IPV and NPSV perpetration, and women's experience of IPV and NPSV, and key covariates such as alcohol use. This addresses the limitation of many previous studies, which tend to focus on IPV only, and use differing measures of drivers and outcomes examining the associations between IPV and NPSV and mental health. The study also adds to the scant evidence base in the global South, where the co-occurring problems of poor mental health and VAWG are prevalent. Furthermore, it highlights the complex and overlapping nature of alcohol, poor mental health as drivers and outcomes of VAWG, often against the backdrop of other structural drivers, such as poverty, food insecurity and unemployment. However, we also recognize limitations in the research. Previous research suggests that there is likely a bi-directional relationship between recent IPV and poor mental health particularly for women (10, 42). However, the cross-sectional nature of the current data limits our ability to draw any conclusions about the temporal relationships, and necessitates future longitudinal analysis. Only two of the five studies were population-based and other studies were limited in generalizability, as they were based on populations recruited for the purpose of impact evaluation. Despite the current limitations, the study confirms that poor mental health are important drivers of men's use of violence, and women experiencing IPV and NPSV often experience poor

For peer review only - http://bmjopen.bmj.com/site/about/guidelines.xhtml

mental health in LIMIC settings. We recognise that the study only has two measures of mental health symptoms. There may be previous psychiatric history accounting particularly for men's perpetration of both IPV and NPSV. However, we do not have data on a wider range of psychiatric symptomology from the current studies in these LMIC settings and, future studies examining risk factors for IPV and NPSV perpetration should take these into account.

Conclusion

Our analysis has shown a consistent pattern of depression and PTSD symptoms as drivers of men's perpetration of IPV and NPSV, and increased depression and PTSD symptoms as outcomes among women who are in relationships characterised by IPV and NPSV. Interventions to address VAWG should more actively consider addressing poor mental health as drivers, and address poor mental health outcomes among women experiencing IPV and NPSV in LMIC settings in prevention and treatment programmes.

Acknowledgements

The authors wish to thank the research teams, programme staff, and participants from all studies included in this pooled analysis.

Contributorship statement

LR conceptualized the analysis with AG and RJ. EC led the statistical analysis. AG was a PI on the Stepping Stones and Creating Futures study in South Africa, EC and DOA, and AAL were Co- PIs on the RRS-COMBAT study in Ghana, KD was a PI on the Indashyikirwa study in Rwanda, and MM is a VAWG and mental health researcher, who contributed to the overall drafting of the manuscript. RJ was the Director of the overall What Works to Prevent Violence Against Women and Girls Global programme. LR led the drafting of the manuscript; all authors contributed to comments and revisions.

Funding

All studies presented and this pooled analysis of the data were funded through the What Works to Prevent Violence? A Global Programme on Violence Against Women and Girls (VAWG) funded by the UK Government's Department for International Development (DFID), Grant number: PO 6254. However, the views expressed do not necessarily reflect the department's official policies and the funders had no role in study design; collection, management, analysis, and interpretation of data; writing of the report; and the decision to submit the paper for publication. Funding was managed by the South African Medical

For peer review only - http://bmjopen.bmj.com/site/about/guidelines.xhtml

Research Council. Time drafting this manuscript was funded by the South African Medical Research Council.

Competing Interest

There are no potential conflicts of interest to declare.

Data sharing

De- identified individual participant data for Stepping Stones and Creating Futures (South Africa), Sonke Change trial (South Africa), and Evaluation of the RRS-COMBAT intervention (Ghana) and oPt intervention , are available to anyone who wishes to access the data for any purpose at https://medat.samrc.ac.za/index.php/catalog/WW . De- identified individual participant data from the Indashyikirwa couples surveys (Rwanda) are available from the Principal Investigator of the study, Dr Kristin Dunkle: kristin.dunkle@mrc.ac.za , but may require permission from the Rwandan Ministry of Gender and Family Promotion (MIGEPROF) before transfer.

ORCID iD

Leane Ramsoomar: http://orcid.org/0000-0003-1934-579X

Andrew Gibbs: http://orcid.org/0000-0003-2812-5377

Esnat Chirwa: http://orcid.org/ 0000-0003-0471-4978

Kristin Dunkle: http://orcid.org/0000-0003-3071-5544

Rachel Jewkes http://orcid.org/0000-0002-4330-6267

Mercilene Machisa https://orcid.org/0000-0001-7275-1100

Deda Ogum-Alangea http://orcid.org/0000-0001-7496-8514

Adolphina Addo-Lartey https://orcid.org/0000-0001-8852-7189

Ethical issues

Ethical clearance for all studies was obtained prior to the studies commencing. For the Stepping Stones and Creating Futures Trial, clearance was obtained from the South African Medical Research Council's Ethics Committee (EC006-2/2015) and the University of KwaZulu-Natal’s Biomedical Research Ethics Committee (BFC043/15). For the Sonke Change Trial, ethical clearance was obtained from the University

For peer review only - http://bmjopen.bmj.com/site/about/guidelines.xhtml

of Witwatersrand's Ethics Committee (M150443). For the Indashyikirwa couple's intervention in Rwanda, ethical approval was obtained from the Rwandan National Ethics Committee (340/RNEC/2015) and the South Africa Medical Research Council Ethics Committee (EC033-10/2015). A required research permit was obtained from the National Institute of Statistics Rwanda (0738/2015/10/NISR). For the RRS-COMBAT intervention in Ghana, the Noguchi Memorial Institute for Medical Research, University of Ghana ((# 006/15–16) and the South African Medical Research Council's Ethics Committee (EC031-9/2015) granted ethical clearance for the study. Ethical clearance for the oPt study was granted by the South African Medical Research Council's ethics committee (EC014-5/2016). All studies followed ethical and safety guidelines for research on violence against women. All participants provided written informed consent before participation. Further information on the studies ethics is available in the study sources referenced in Table 1.

For peer review only

References

1. World Health Organization. Violence against women prevalence estimates, 2018: global, regional and national prevalence estimates for intimate partner violence against women and global and regional prevalence estimates for non-partner sexual violence against women. 2021.
2. Ellsberg M, Jansen HA, Heise L, Watts CH, Garcia-Moreno C. Intimate partner violence and women's physical and mental health in the WHO multi-country study on women's health and domestic violence: an observational study. The lancet. 2008;371(9619):1165-72.
3. Gibbs A, Dunkle K, Ramsoomar L, Willan S, Jama Shai N, Chatterji S, et al. New learnings on drivers of men's physical and/or sexual violence against their female partners, and women's experiences of this, and the implications for prevention interventions. Global health action. 2020;13(1):1739845.
4. Abramsky T, Watts CH, Garcia-Moreno C, Devries K, Kiss L, Ellsberg M, et al. What factors are associated with recent intimate partner violence? Findings from the WHO multi-country study on women's health and domestic violence. BMC public health. 2011;11(1):109.
5. Ellsberg M, Ovince J, Murphy M, Blackwell A, Reddy D, Stennes J, et al. No safe place: Prevalence and correlates of violence against conflict-affected women and girls in South Sudan. PLoS one. 2020;15(10):e0237965.
6. Stöckl H, Sardinha L, Maheu-Giroux M, Meyer SR, García-Moreno C. Physical, sexual and psychological intimate partner violence and non-partner sexual violence against women and girls: a systematic review protocol for producing global, regional and country estimates. BMJ open. 2021;11(8):e045574.
7. Machisa MT, Christofides N, Jewkes R. Mental ill health in structural pathways to women's experiences of intimate partner violence. PloS one. 2017;12(4):e0175240.
8. Machisa MT, Christofides N, Jewkes R. Structural pathways between child abuse, poor mental health outcomes and male-perpetrated intimate partner violence (IPV). PloS one. 2016;11(3):e0150986.
9. Lagdon S, Armour C, Stringer M. Adult experience of mental health outcomes as a result of intimate partner violence victimisation: a systematic review. European journal of psychotraumatology. 2014;5(1):24794.
10. Bacchus LJ, Ranganathan M, Watts C, Devries K. Recent intimate partner violence against women and health: a systematic review and meta-analysis of cohort studies. BMJ open. 2018;8(7):e019995.
11. Devries KM, Mak JY, Bacchus LJ, Child JC, Falder G, Petzold M, et al. Intimate partner violence and incident depressive symptoms and suicide attempts: a systematic review of longitudinal studies. PLoS medicine. 2013;10(5):e1001439.
12. Bell KM, Orcutt HK. Posttraumatic stress disorder and male-perpetrated intimate partner violence. JAmA. 2009;302(5):562-4.
13. Orcutt HK, King LA, King DW. Male-perpetrated violence among Vietnam veteran couples: relationships with veteran's early life characteristics, trauma history, and PTSD symptomatology. Journal of traumatic stress. 2003;16(4):381-90.
14. Trevillion K, Williamson E, Thandi G, Borschmann R, Oram S, Howard LM. A systematic review of mental disorders and perpetration of domestic violence among military populations. Social psychiatry and psychiatric epidemiology. 2015;50(9):1329-46.
15. Jordan BK, Marmar CR, Fairbank JA, Schlenger WE, Kulka RA, Hough RL, et al. Problems in families of male Vietnam veterans with posttraumatic stress disorder. Journal of consulting and clinical psychology. 1992;60(6):916.
16. King LA, King DW. Male-perpetrated domestic violence: Testing a series of multifactorial family models. Violence Against Women and Family Violence: Developments in Research, Practice, and Policy. 2004.

For peer review only - http://bmjopen.bmj.com/site/about/guidelines.xhtml

17. Fulu E, Jewkes R, Roselli T, Garcia-Moreno C. Prevalence of and factors associated with male perpetration of intimate partner violence: findings from the UN Multi-country Cross-sectional Study on Men and Violence in Asia and the Pacific. The lancet global health. 2013;1(4):e187-e207.
18. Machisa MT. The relationships between poor mental health, child abuse, women's experiences and male perpetration of intimate partner violence in Gauteng province of South Africa. Johannesburg: University of the Witwatersrand; 2019.
19. O'Donnell ML, Creamer M, Pattison P. Posttraumatic stress disorder and depression following trauma: understanding comorbidity. American Journal of Psychiatry. 2004;161(8):1390-6.
20. Hatcher AM, Gibbs A, Jewkes R, McBride R-S, Peacock D, Christofides N. Effect of childhood poverty and trauma on adult depressive symptoms among young men in peri-urban South African settlements. Journal of Adolescent Health. 2019;64(1):79-85.
21. Ellsberg M, Heise L, Pena R, Agurto S, Winkvist A. Researching domestic violence against women: methodological and ethical considerations. Studies in family planning. 2001;32(1):1-16.
22. Radloff LS. A self-report depression scale for research in the general population. Applied psychol Measurements. 1977;1:385-401.
23. Blodgett JM, Lachance CC, Stubbs B, Co M, Wu Y-T, Prina M, et al. A systematic review of the latent structure of the Center for Epidemiologic Studies Depression Scale (CES-D) amongst adolescents. BMC psychiatry. 2021;21(1):1-8.
24. Mollica RF, Caspi-Yavin Y, Bollini P, Truong T, Tor S, Lavelle J. The Harvard Trauma Questionnaire: validating a cross-cultural instrument for measuring torture, trauma, and posttraumatic stress disorder in Indochinese refugees. Journal of nervous and mental disease. 1992.
25. Darzil C. The Harvard Trauma Questionnaire: Reliability and Validity Generalization Studies of the Symptom Scales. Canada: University of Ottawa; 2017.
26. Christofides NJ, Hatcher AM, Pino A, Rebombo D, McBride RS, Anderson A, et al. A cluster randomised controlled trial to determine the effect of community mobilisation and advocacy on men's use of violence in periurban South Africa: study protocol. BMJ open. 2018;8(3):e017579.
27. World Health Organization. International guide for monitoring alcohol consumption and related harm. World Health Organization; 2000.
28. Chen CM, Dufour MC, Yi H-y. Alcohol consumption among young adults ages 18–24 in the United States: Results from the 2001–2002 NESARC survey. Alcohol Research & Health. 2004;28(4):269.
29. Tevik K, Bergh S, Selbæk G, Johannessen A, Helvik A-S. A systematic review of self-report measures used in epidemiological studies to assess alcohol consumption among older adults. PloS one. 2021;16(12):e0261292.
30. García-Moreno C, Jansen HA, Ellsberg M, Heise L, Watts C. WHO multi-country study on women's health and domestic violence against women: World Health Organization; 2005.
31. Jewkes R, Dunkle K, Koss MP, Levin JB, Nduna M, Jama N, et al. Rape perpetration by young, rural South African men: Prevalence, patterns and risk factors. Social science & medicine. 2006;63(11):2949-61.
32. Jewkes R, Fulu E, Tabassam Naved R, Chirwa E, Dunkle K, Haardörfer R, et al. Women's and men's reports of past-year prevalence of intimate partner violence and rape and women's risk factors for intimate partner violence: A multicountry cross-sectional study in Asia and the Pacific. PLoS medicine. 2017;14(9):e1002381.
33. Voith LA, Lee H, Russell K. How Trauma, Depression, and Gender Roles Lead to Intimate Partner Violence Perpetration Among a Sample of Predominately Low-Income Black, Indigenous, Men of Color: A Mixed Methods Study. Journal of Interpersonal Violence. 2022;37(9-10):NP6487-NP513.
34. Stith SM, Smith DB, Penn CE, Ward DB, Tritt D. Intimate partner physical abuse perpetration and victimization risk factors: A meta-analytic review. Aggression and violent behavior. 2004;10(1):65-98.

For peer review only - http://bmjopen.bmj.com/site/about/guidelines.xhtml

35. Schumacher JA, Feldbau-Kohn S, Slep AMS, Heyman RE. Risk factors for male-to-female partner physical abuse. Aggression and violent behavior. 2001;6(2-3):281-352.
36. Shorey RC, Febres J, Brasfield H, Stuart GL. The prevalence of mental health problems in men arrested for domestic violence. Journal of family violence. 2012;27(8):741-8.
37. Ramsoomar L, Gibbs A, Chirwa ED, Dunkle K, Jewkes R. Pooled analysis of the association between alcohol use and violence against women: evidence from four violence prevention studies in Africa. BMJ open. 2021;11(7):e049282.
38. Gibbs A, Govender K, Jewkes R. An exploratory analysis of factors associated with depression in a vulnerable group of young people living in informal settlements in South Africa. Global public health. 2018;13(7):788-803.
39. Riggs DS, Caulfield MB, Street AE. Risk for domestic violence: Factors associated with perpetration and victimization. Journal of clinical psychology. 2000;56(10):1289-316.
40. Jewkes R, Jama-Shai N, Sikweyiya Y. Enduring impact of conflict on mental health and gender-based violence perpetration in Bougainville, Papua New Guinea: a cross-sectional study. Plos one. 2017;12(10):e0186062.
41. Dixon LB, Holoshitz Y, Nossel I. Treatment engagement of individuals experiencing mental illness: review and update. World Psychiatry. 2016;15(1):13-20.
42. Devries KM, Child JC, Bacchus LJ, Mak J, Falder G, Graham K, et al. Intimate partner violence victimization and alcohol consumption in women: A systematic review and meta-analysis. Addiction. 2014;109(3):379-91.

For peer review only

For peer review only

For peer review only - http://bmjopen.bmj.com/site/about/guidelines.xhtml

STROBE 2007 (v4) Statement—Checklist of items that should be included in reports of *cross-sectional studies*

Section/Topic	Item #	Recommendation	Reported on page #
**Title and abstract**	1	(*a*) Indicate the study's design with a commonly used term in the title or the abstract	Page 1
		(*b*) Provide in the abstract an informative and balanced summary of what was done and what was found	Page 2, Abstract
**Introduction**			
Background/rationale	2	Explain the scientific background and rationale for the investigation being reported	Pages 4-6
Objectives	3	State specific objectives, including any prespecified hypotheses	Page 6, lines 4-6
**Methods**			
Study design	4	Present key elements of study design early in the paper	Page 2, line 6; Page 5, line 30
Setting	5	Describe the setting, locations, and relevant dates, including periods of recruitment, exposure, follow-up, and data collection	Page 6, Methods, Lines 12-21
Participants	6	(*a*) Give the eligibility criteria, and the sources and methods of selection of participants	Page7 , Table 1 (Pooled analysis of five studies)
Variables	7	Clearly define all outcomes, exposures, predictors, potential confounders, and effect modifiers. Give diagnostic criteria, if applicable	Page 8 (Measures) Pages 9-10 (Table 2 Key measures) Page 11, Lines 19-25
Data sources/ measurement	8*	For each variable of interest, give sources of data and details of methods of assessment (measurement). Describe comparability of assessment methods if there is more than one group	Page 8 (Measures) Pages 9-10 (Table 2 Key measures)
Bias	9	Describe any efforts to address potential sources of bias	Page 11, data analysis, lines 15-19
Study size	10	Explain how the study size was arrived at	This was a pooled analysis of data from 5 studies . The sample size in the

For peer review only

			pooled analysis totalled 7021 men and 4,525 women 18+ years old from a mix of self-selecting and randomly selected household surveys. (see Page 7, Table 1)
Quantitative variables	11	Explain how quantitative variables were handled in the analyses. If applicable, describe which groupings were chosen and why	Pages 9-10, Table 2, Key Measures and Page 11, data analysis
Statistical methods	12	(*a*) Describe all statistical methods, including those used to control for confounding	Page 11, data analysis
		(*b*) Describe any methods used to examine subgroups and interactions	Page 11, data analysis
		(*c*) Explain how missing data were addressed	Page 11, data analysis
		(*d*) If applicable, describe analytical methods taking account of sampling strategy	Page 11, data analysis
		(*e*) Describe any sensitivity analyses	Page 11, data analysis, lines 20-25 and Supplementary Table
**Results**			
Participants	13*	(a) Report numbers of individuals at each stage of study—e.g. numbers potentially eligible, examined for eligibility, confirmed eligible, included in the study, completing follow-up, and analysed	Table 1, Page 7 (Baseline data only)
		(b) Give reasons for non-participation at each stage	Baseline data only
		(c) Consider use of a flow diagram	Baseline data only

For peer review only

Descriptive data	14*	(a) Give characteristics of study participants (e.g. demographic, clinical, social) and information on exposures and potential confounders	Pages, 11 and 12<br>Page 13-14, Table 3
		(b) Indicate number of participants with missing data for each variable of interest	
Outcome data	15*	Report numbers of outcome events or summary measures	Page 8-Key measures<br>Pages 9-10, Table 2
Main results	16	(*a*) Give unadjusted estimates and, if applicable, confounder-adjusted estimates and their precision (e.g., 95% confidence interval). Make clear which confounders were adjusted for and why they were included	Page 11, data analysis
		(*b*) Report category boundaries when continuous variables were categorized	
		(*c*) If relevant, consider translating estimates of relative risk into absolute risk for a meaningful time period	
Other analyses	17	Report other analyses done—e.g. analyses of subgroups and interactions, and sensitivity analyses	Page 11, lines 20-25 and Supplementary Table
**Discussion**			
Key results	18	Summarise key results with reference to study objectives	Pages 20-22
Limitations	19	Discuss limitations of the study, taking into account sources of potential bias or imprecision. Discuss both direction and magnitude of any potential bias	Pages 22-23
Interpretation	20	Give a cautious overall interpretation of results considering objectives, limitations, multiplicity of analyses, results from similar studies, and other relevant evidence	Interweaved into Discussion Page 20-22
Generalisability	21	Discuss the generalisability (external validity) of the study results	Page 3, Limitations (Article summary), Page 22 Limitations, line 29
**Other information**			
Funding	22	Give the source of funding and the role of the funders for the present study and, if applicable, for the original study on which the present article is based	Page 23

*Give information separately for cases and controls in case-control studies and, if applicable, for exposed and unexposed groups in cohort and cross-sectional studies.

For peer review only

Note: An Explanation and Elaboration article discusses each checklist item and gives methodological background and published examples of transparent reporting. The STROBE checklist is best used in conjunction with this article (freely available on the Web sites of PLoS Medicine at http://www.plosmedicine.org/, Annals of Internal Medicine at http://www.annals.org/, and Epidemiology at http://www.epidem.com/). Information on the STROBE Initiative is available at www.strobe-statement.org.

For peer review only
